# Supplementary figures and images for: Genetic validation of PfFKBP35 as an antimalarial drug target
Source: eLife. 2023 Nov 7;12:RP86975. doi: 10.7554/eLife.86975 (PMC10629825; doi:10.7554/eLife.86975)

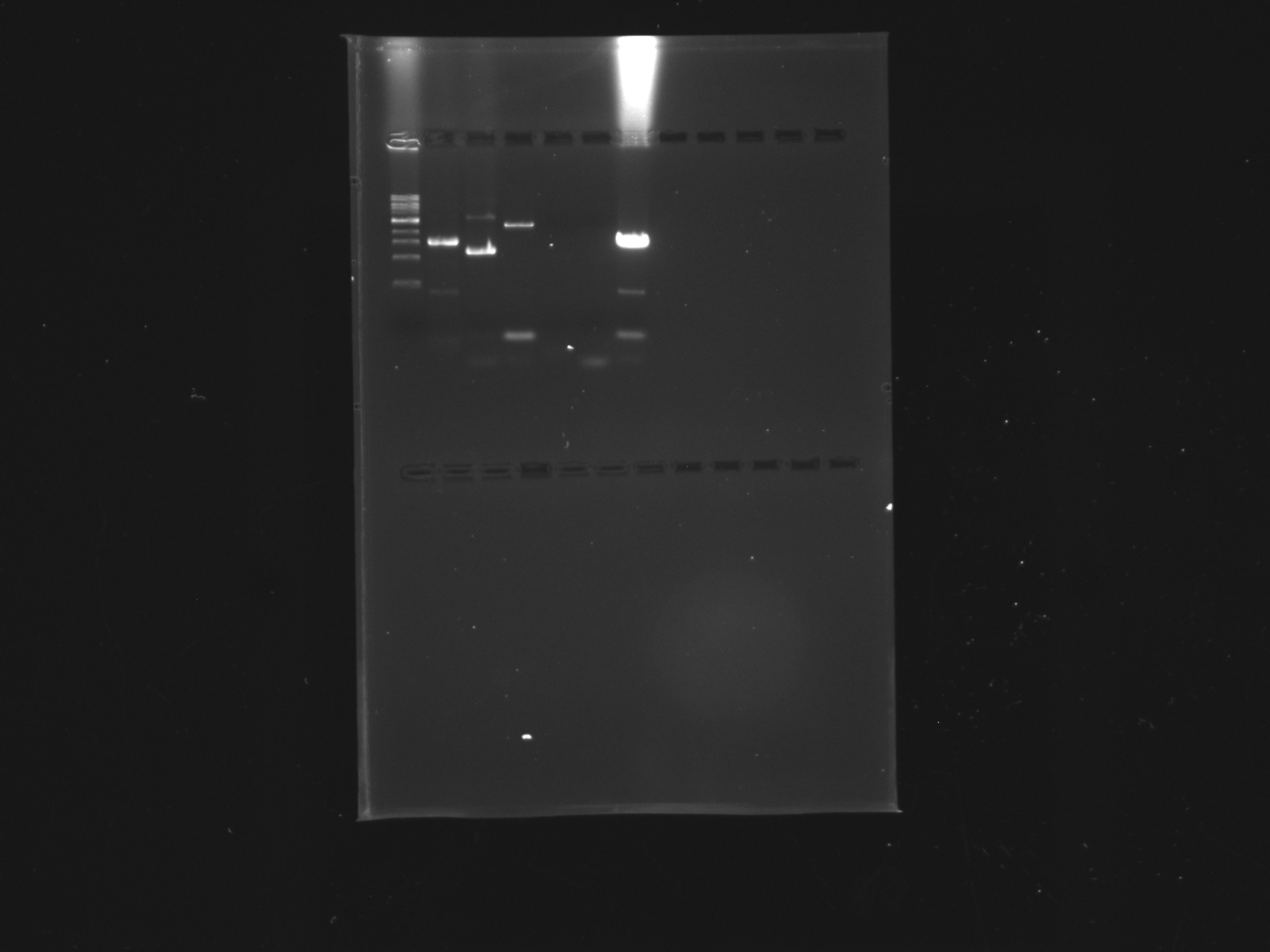

Supplement: Figure 1—figure supplement 1—source data 1. [file elife-86975-fig1-figsupp1-data1.zip › Figure 1-figure supplement 1-source data/Figure 1-figure supplement 1-source data_panel B.Tif]

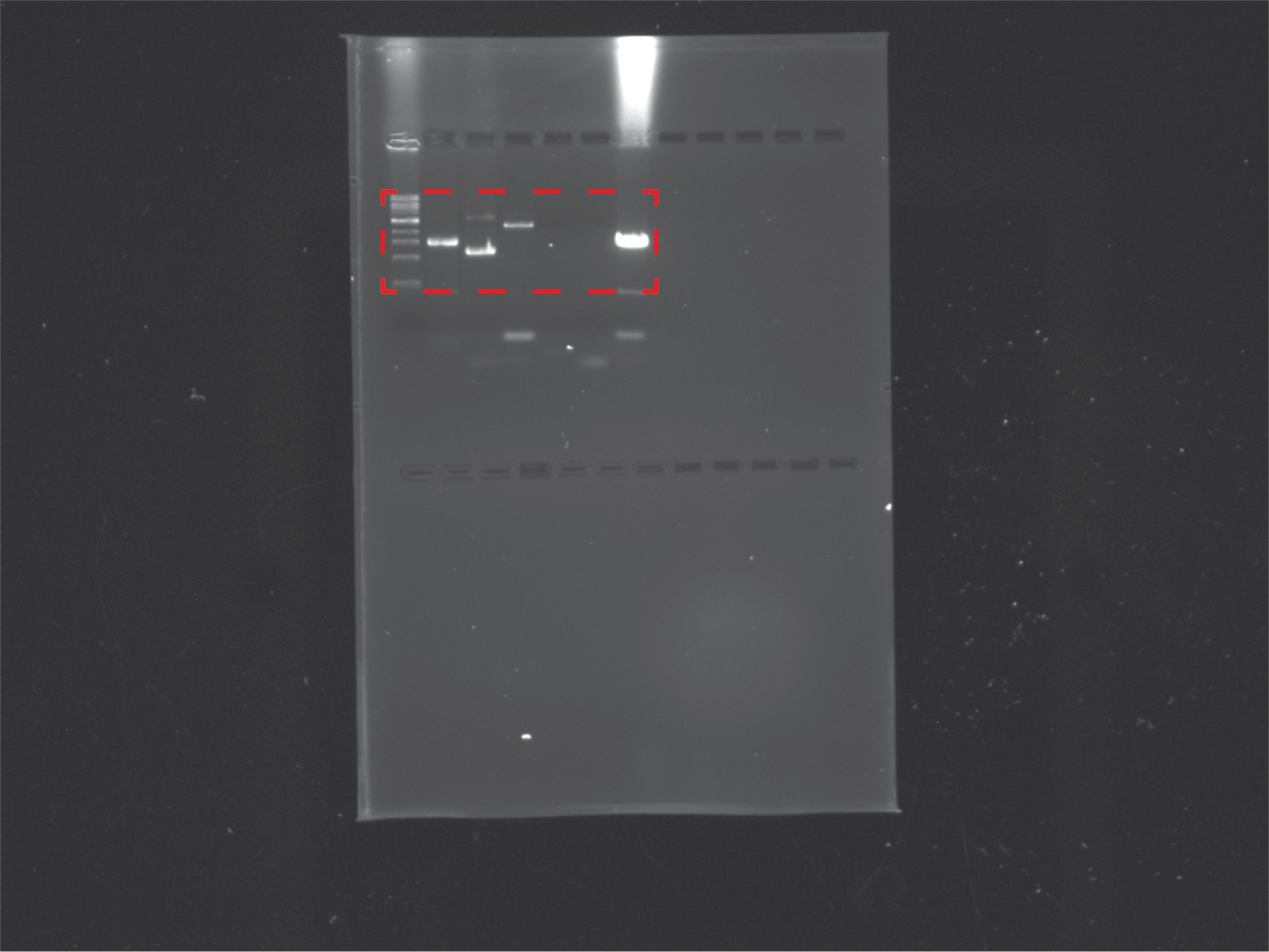

Supplement: Figure 1—figure supplement 1—source data 1. [file elife-86975-fig1-figsupp1-data1.zip › Figure 1-figure supplement 1-source data/Figure 1-figure supplement 1-source data_panel B_labelled.png]

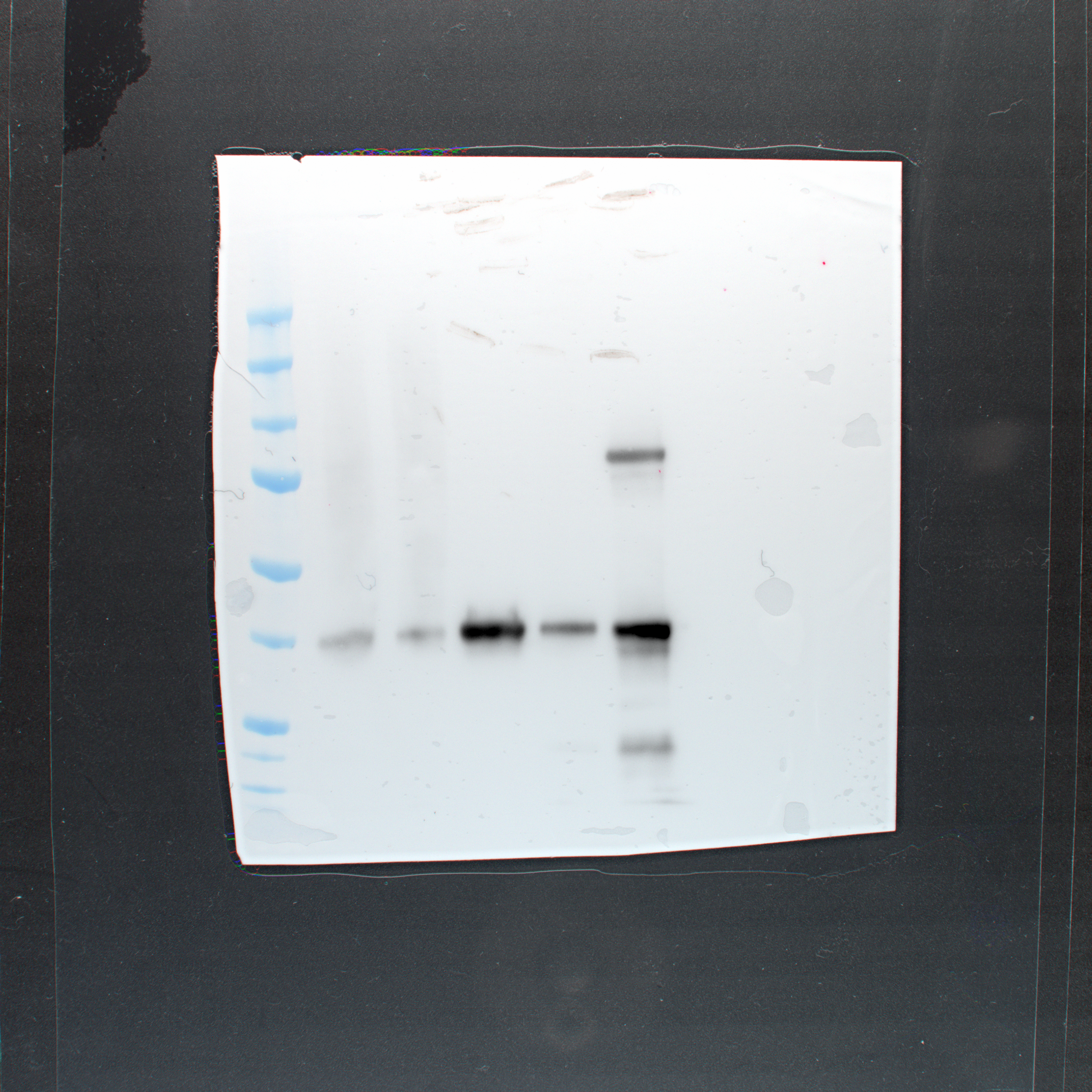

Supplement: Figure 1—figure supplement 1—source data 1. [file elife-86975-fig1-figsupp1-data1.zip › Figure 1-figure supplement 1-source data/Figure 1-figure supplement 1-source data_panel C_a-GAPDH_R1.Tif]

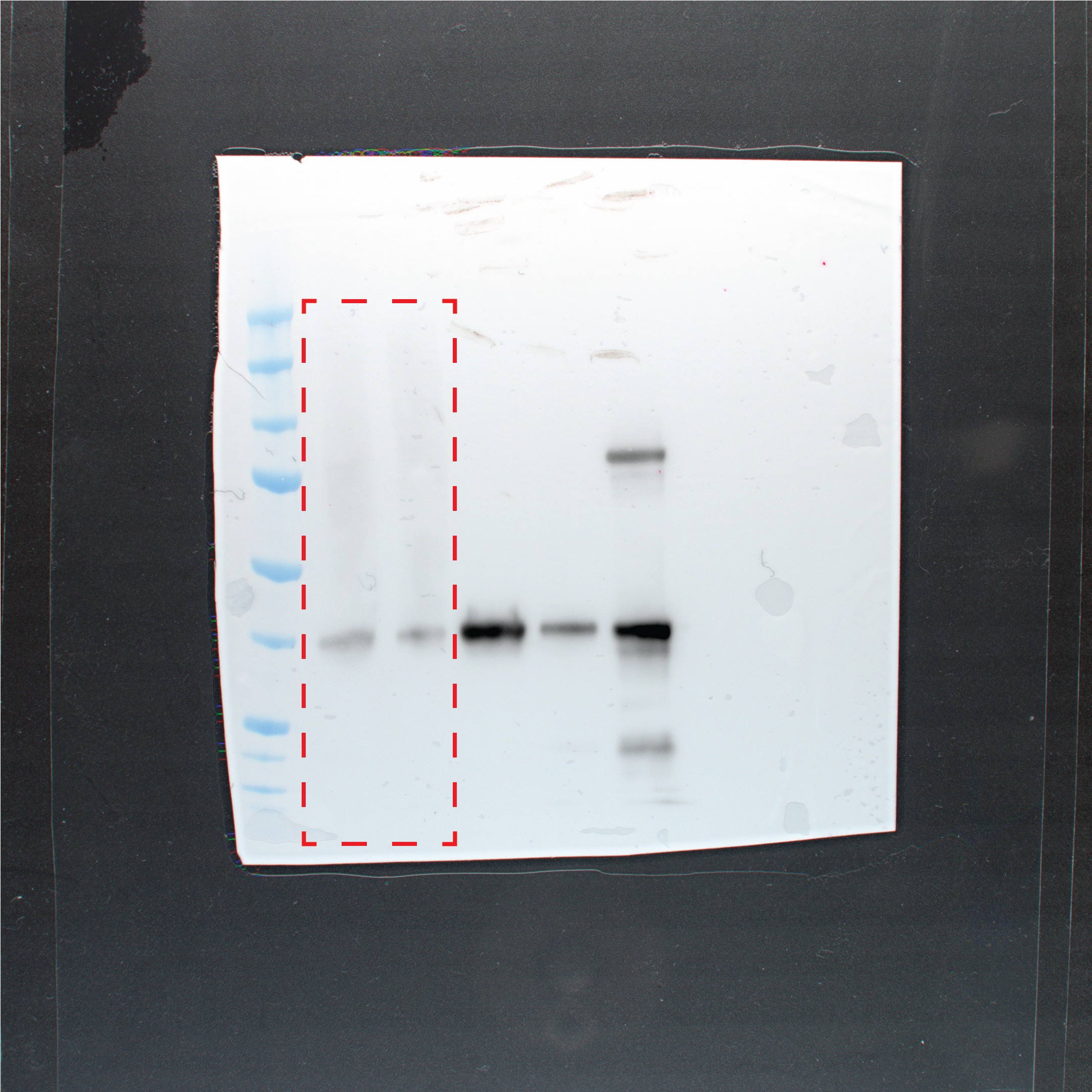

Supplement: Figure 1—figure supplement 1—source data 1. [file elife-86975-fig1-figsupp1-data1.zip › Figure 1-figure supplement 1-source data/Figure 1-figure supplement 1-source data_panel C_a-GAPDH_R1_labelled.png]

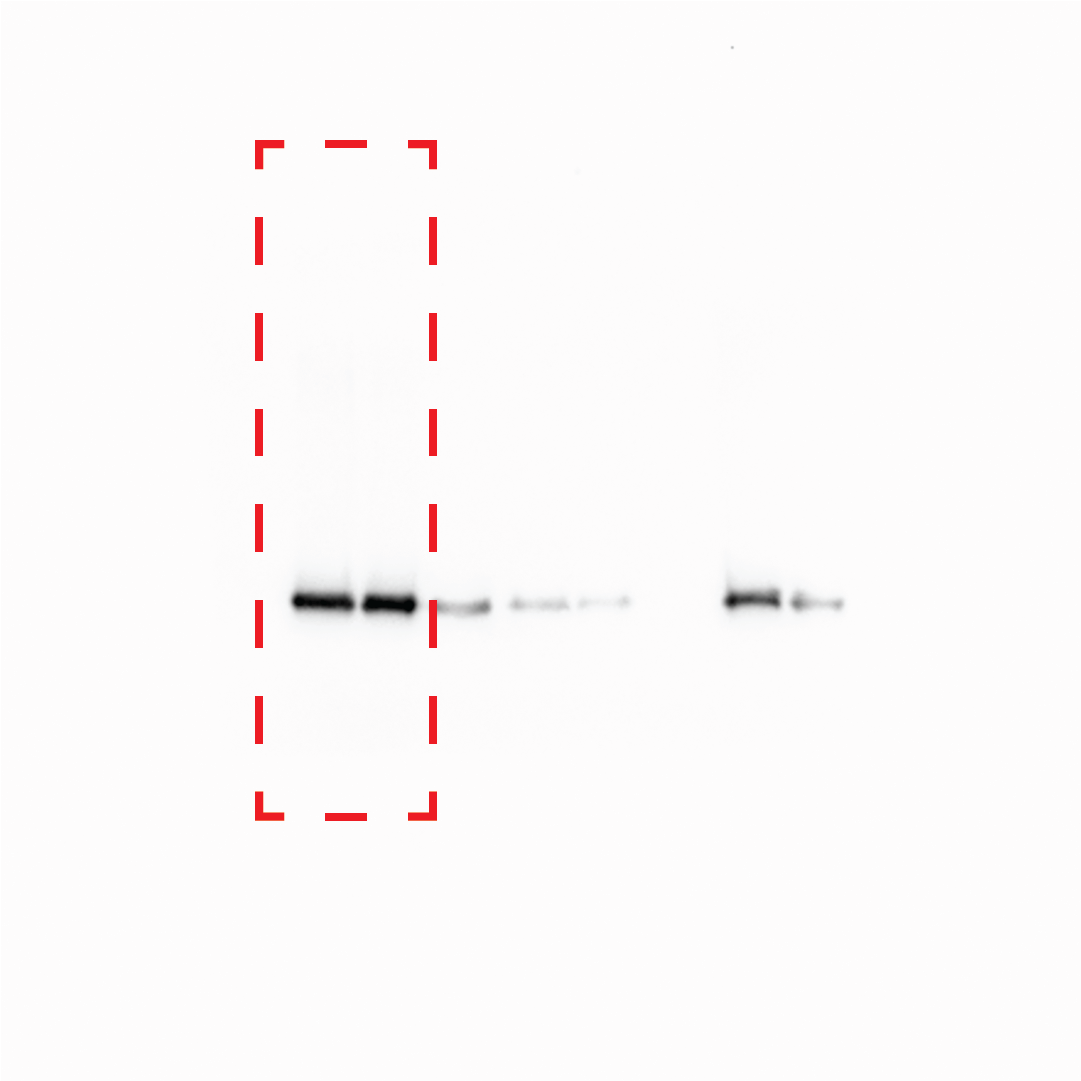

Supplement: Figure 1—figure supplement 1—source data 1. [file elife-86975-fig1-figsupp1-data1.zip › Figure 1-figure supplement 1-source data/Figure 1-figure supplement 1-source data_panel C_a-GAPDH_R2_labelled.png]

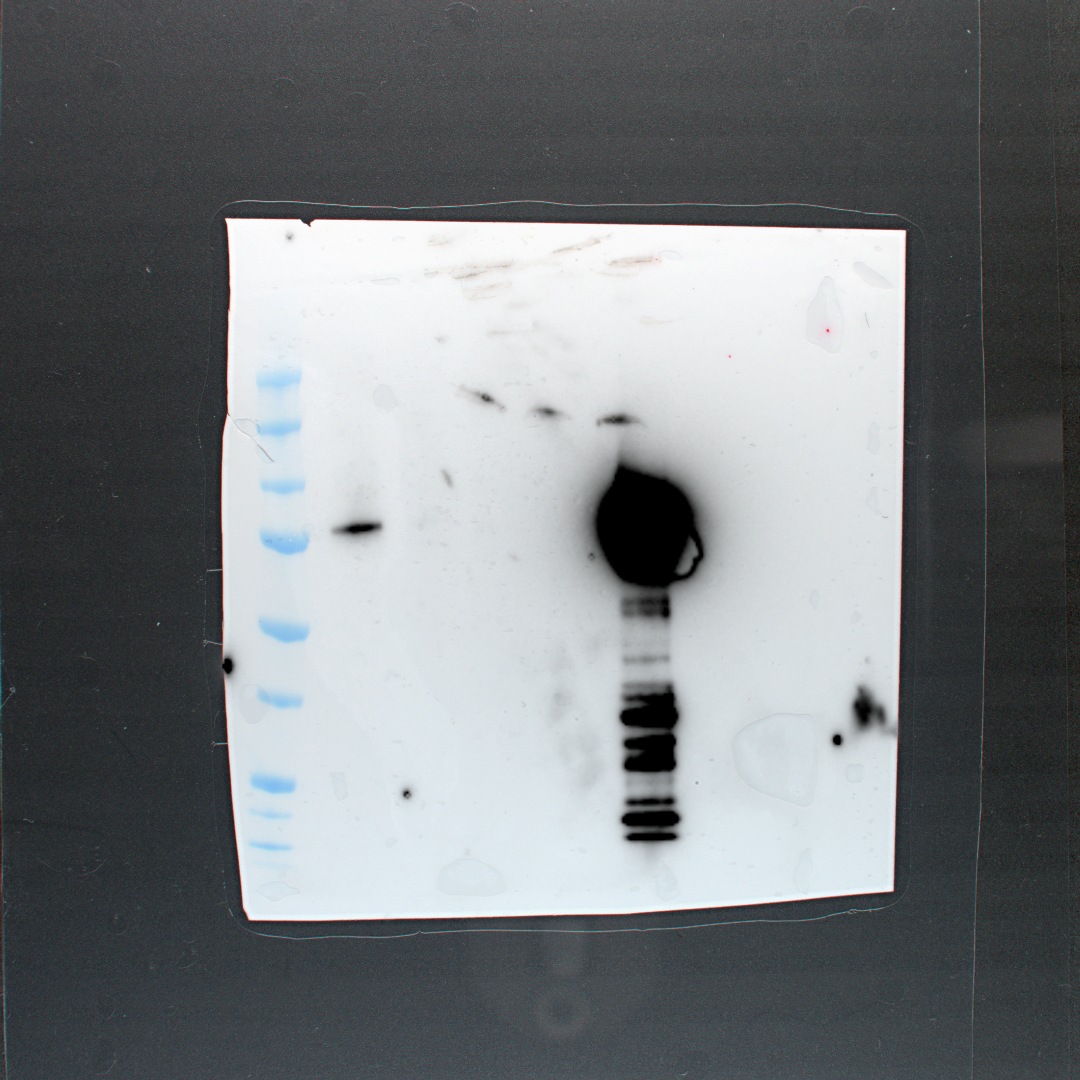

Supplement: Figure 1—figure supplement 1—source data 1. [file elife-86975-fig1-figsupp1-data1.zip › Figure 1-figure supplement 1-source data/Figure 1-figure supplement 1-source data_panel C_a-GFP_R1.Tif]

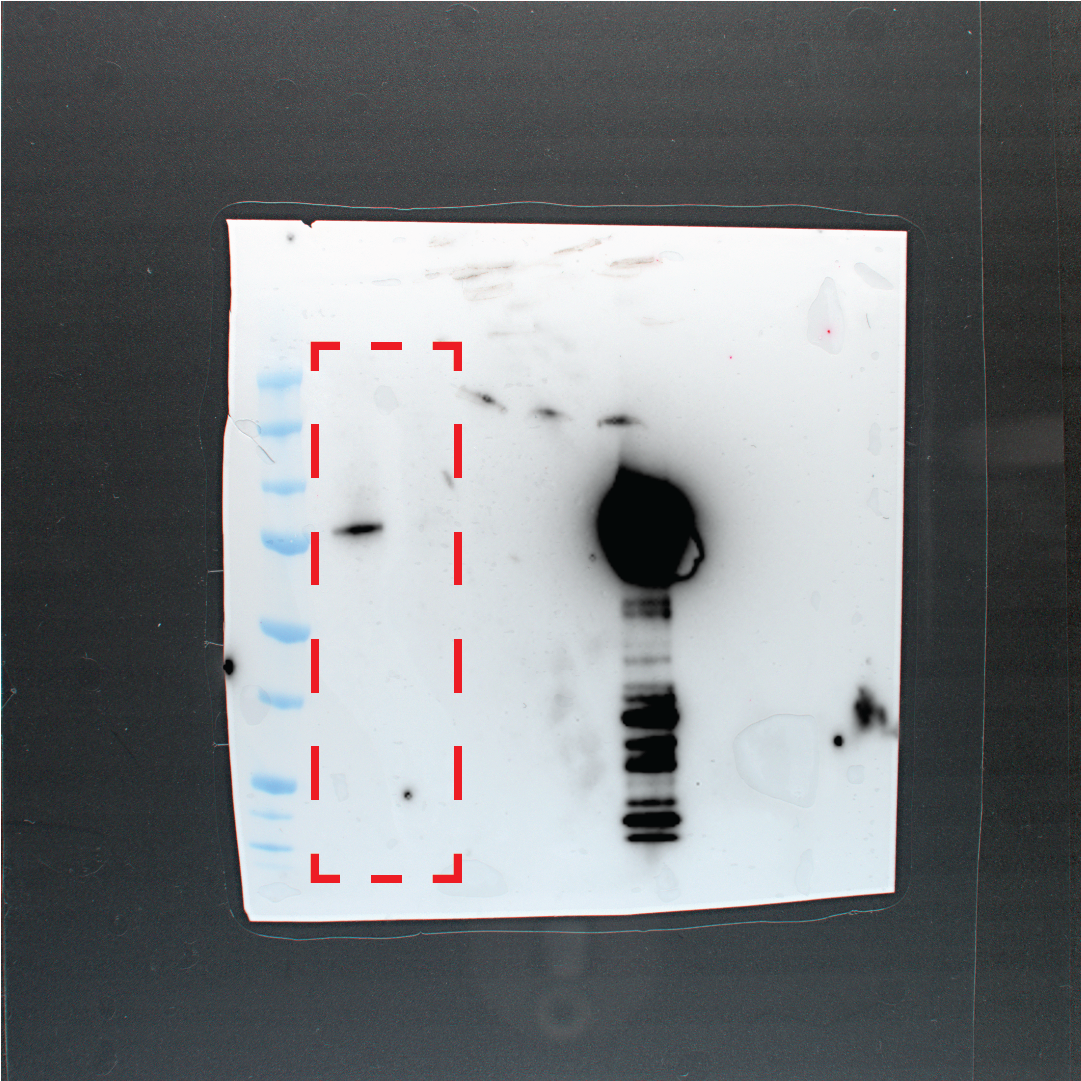

Supplement: Figure 1—figure supplement 1—source data 1. [file elife-86975-fig1-figsupp1-data1.zip › Figure 1-figure supplement 1-source data/Figure 1-figure supplement 1-source data_panel C_a-GFP_R1_labelled.png]

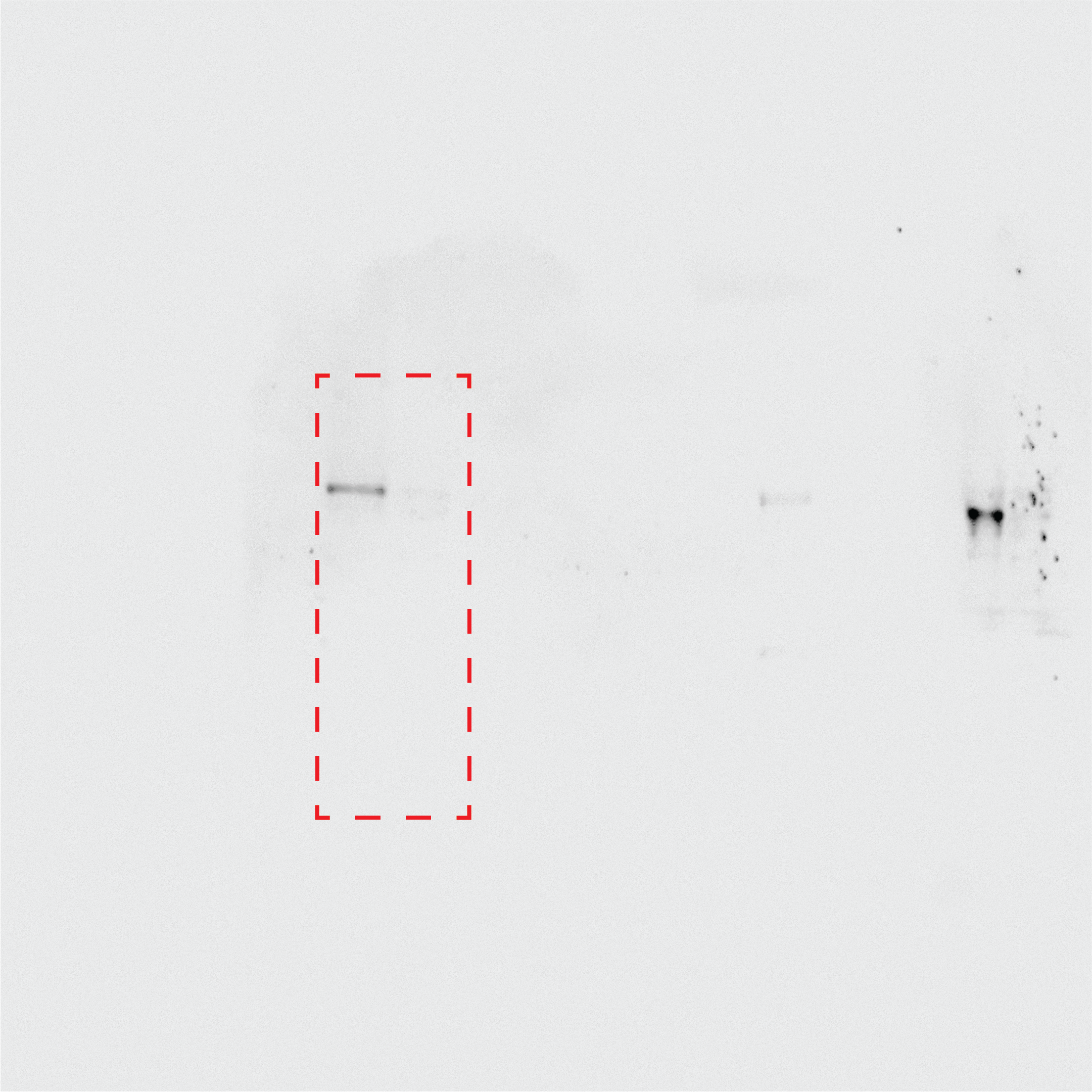

Supplement: Figure 1—figure supplement 1—source data 1. [file elife-86975-fig1-figsupp1-data1.zip › Figure 1-figure supplement 1-source data/Figure 1-figure supplement 1-source data_panel C_a-GFP_R2_labelled.png]

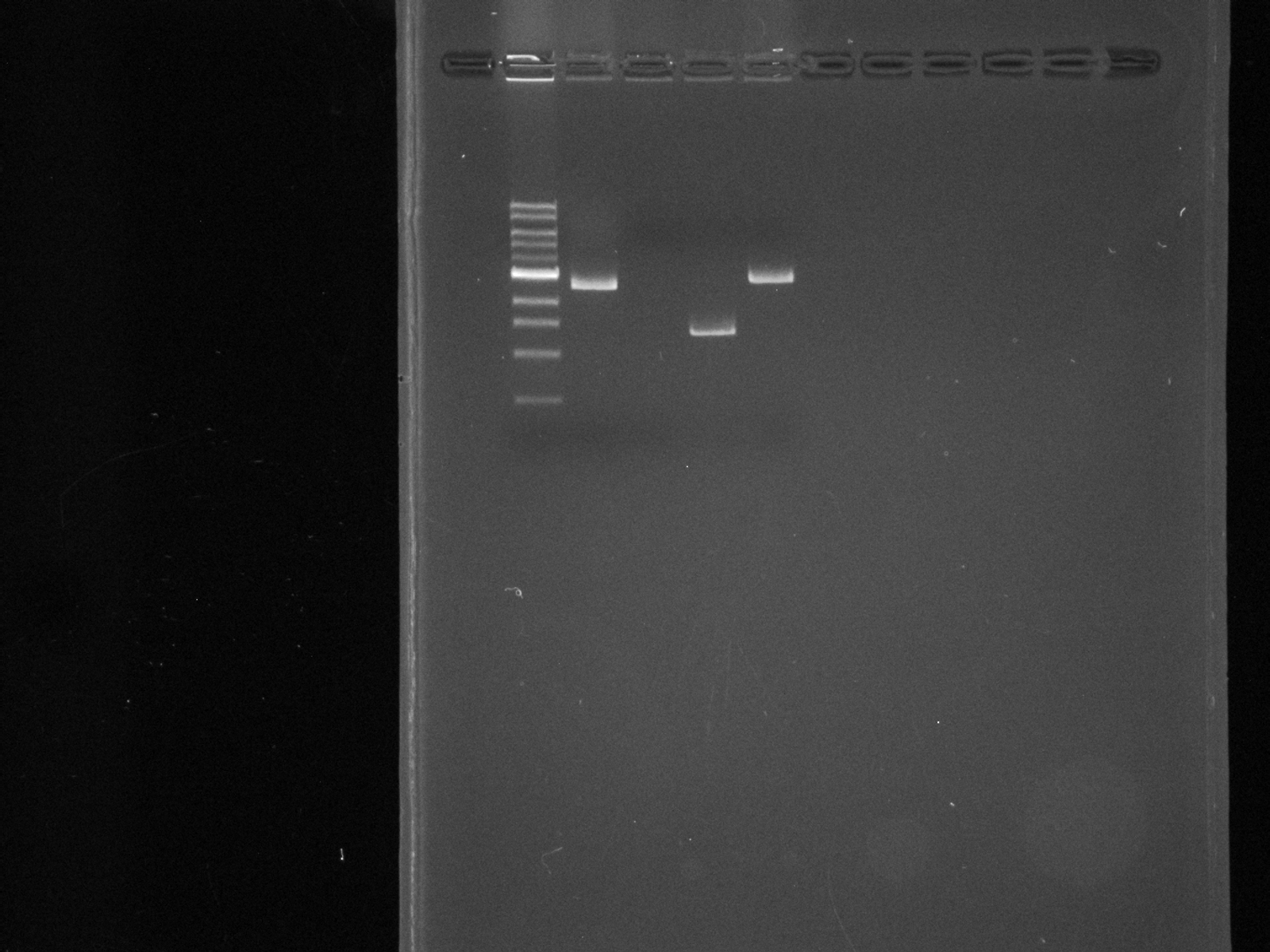

Supplement: Figure 1—figure supplement 2—source data 1. [file elife-86975-fig1-figsupp2-data1.zip › Figure 1-figure supplement 2-source data/Figure 1-figure supplement 2-source data_panel B.Tif]

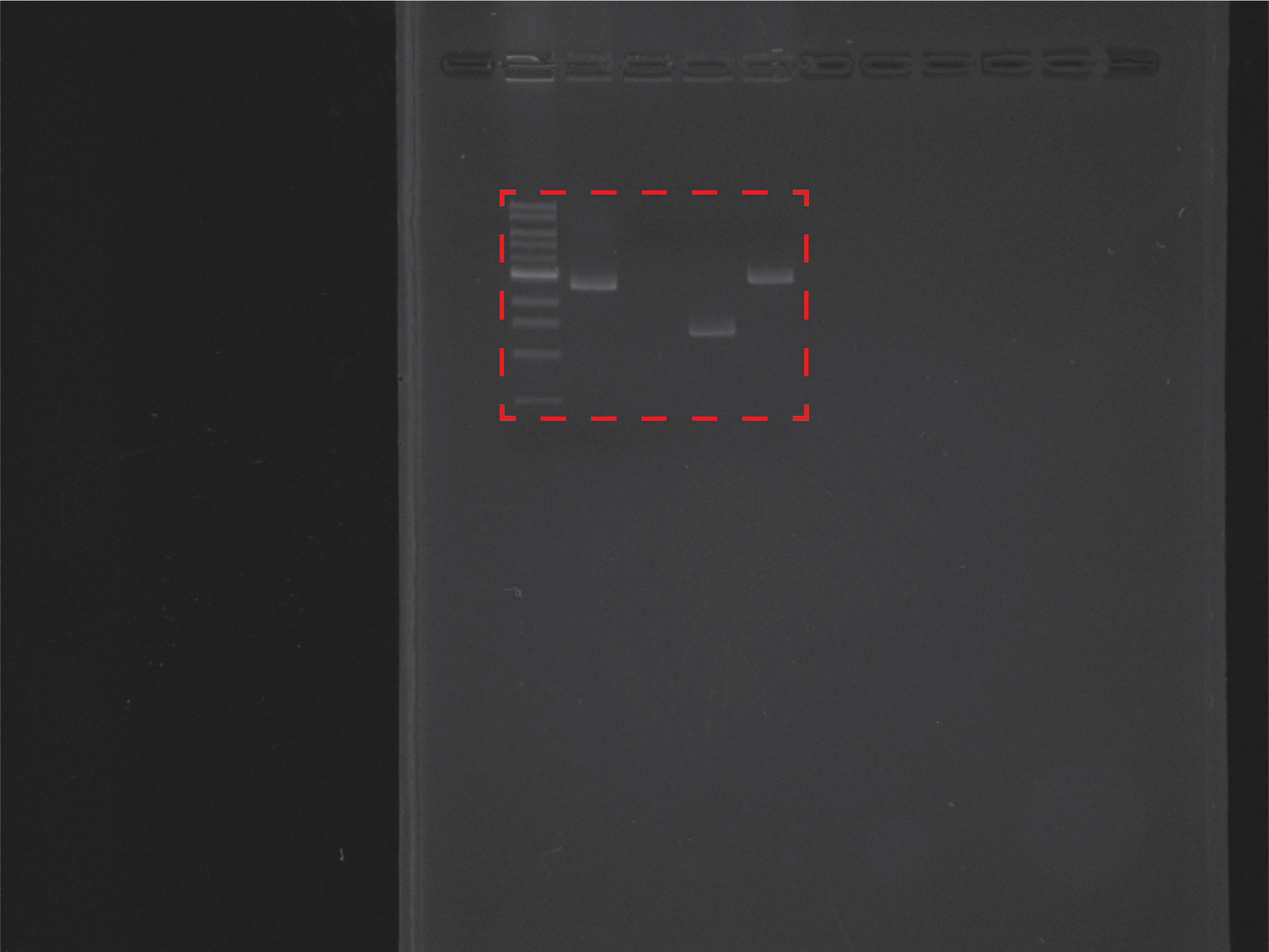

Supplement: Figure 1—figure supplement 2—source data 1. [file elife-86975-fig1-figsupp2-data1.zip › Figure 1-figure supplement 2-source data/Figure 1-figure supplement 2-source data_panel B_labelled.png]

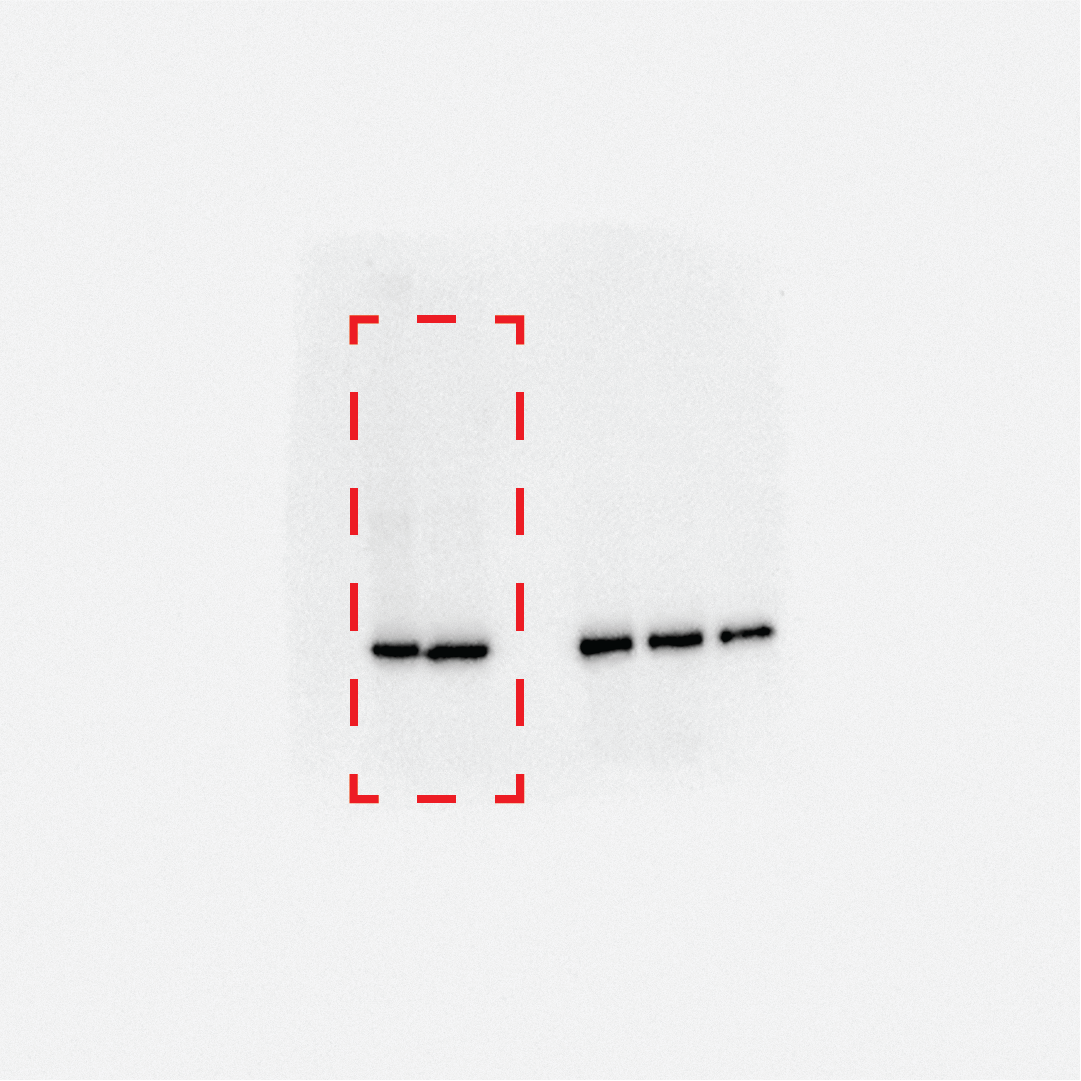

Supplement: Figure 2—source data 1. [file elife-86975-fig2-data1.zip › Figure 2-source data/Figure 2-source data_a-gapdh_labelled.png]

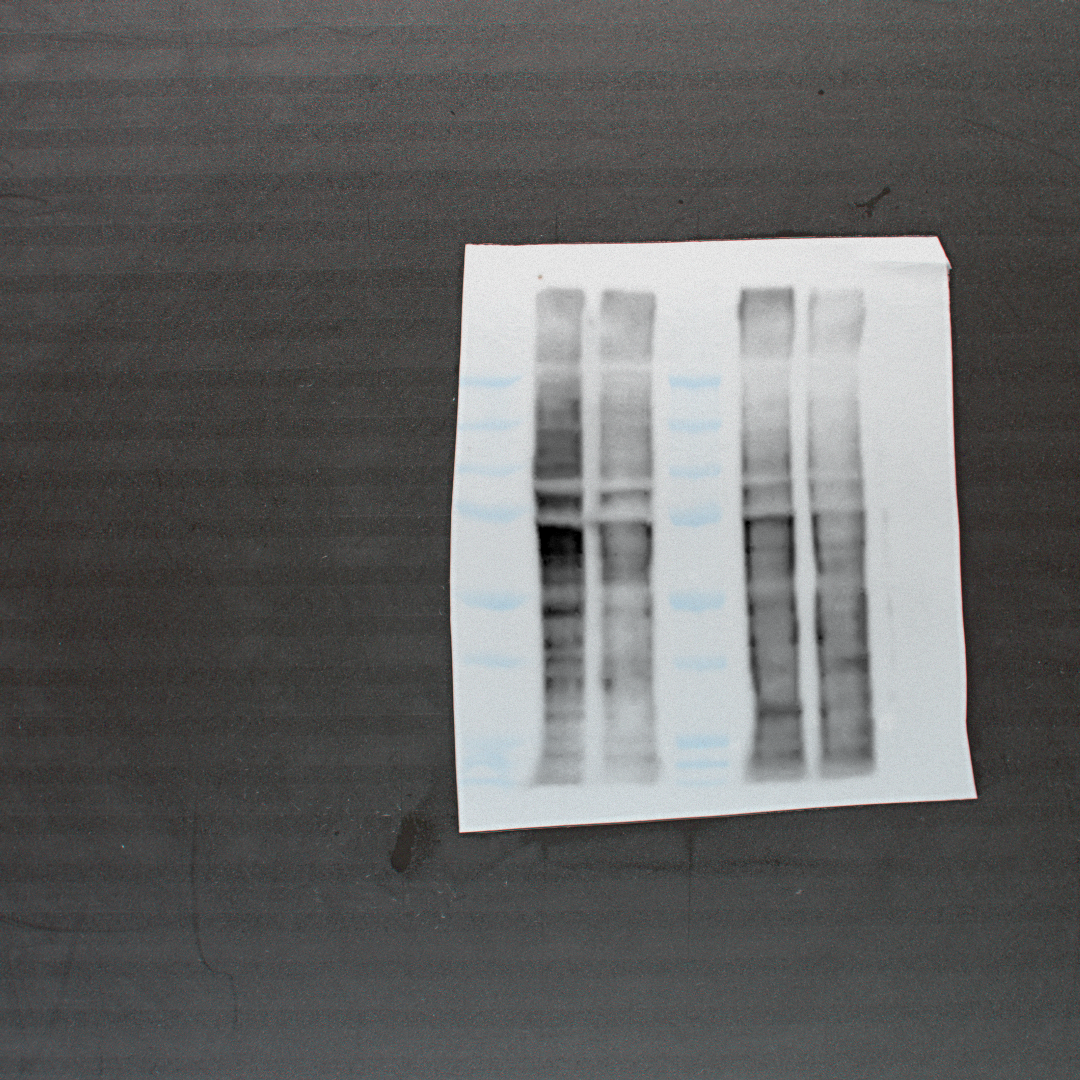

Supplement: Figure 2—source data 1. [file elife-86975-fig2-data1.zip › Figure 2-source data/Figure 2-source data_a-puro.Tif]

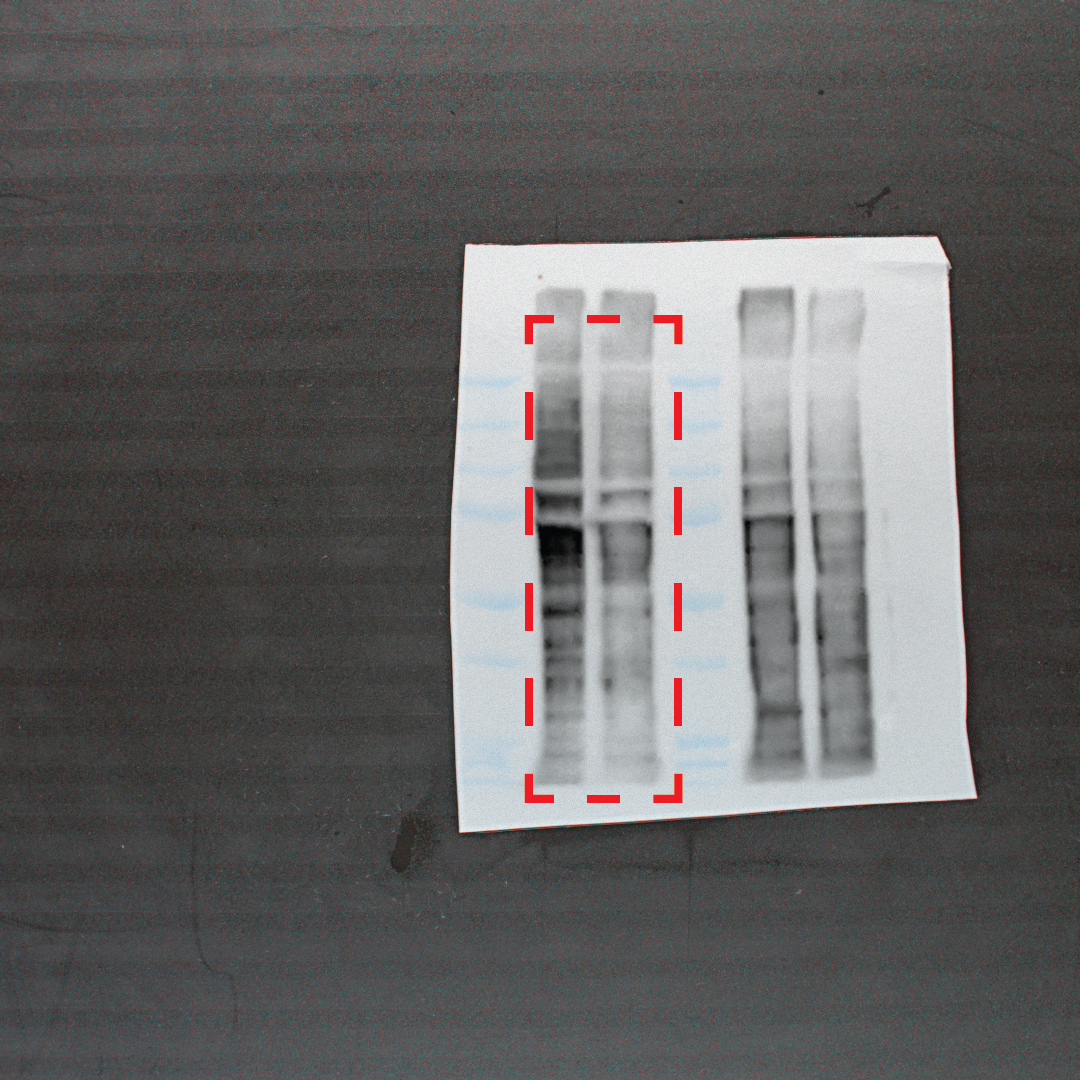

Supplement: Figure 2—source data 1. [file elife-86975-fig2-data1.zip › Figure 2-source data/Figure 2-source data_a-puro_labelled.png]

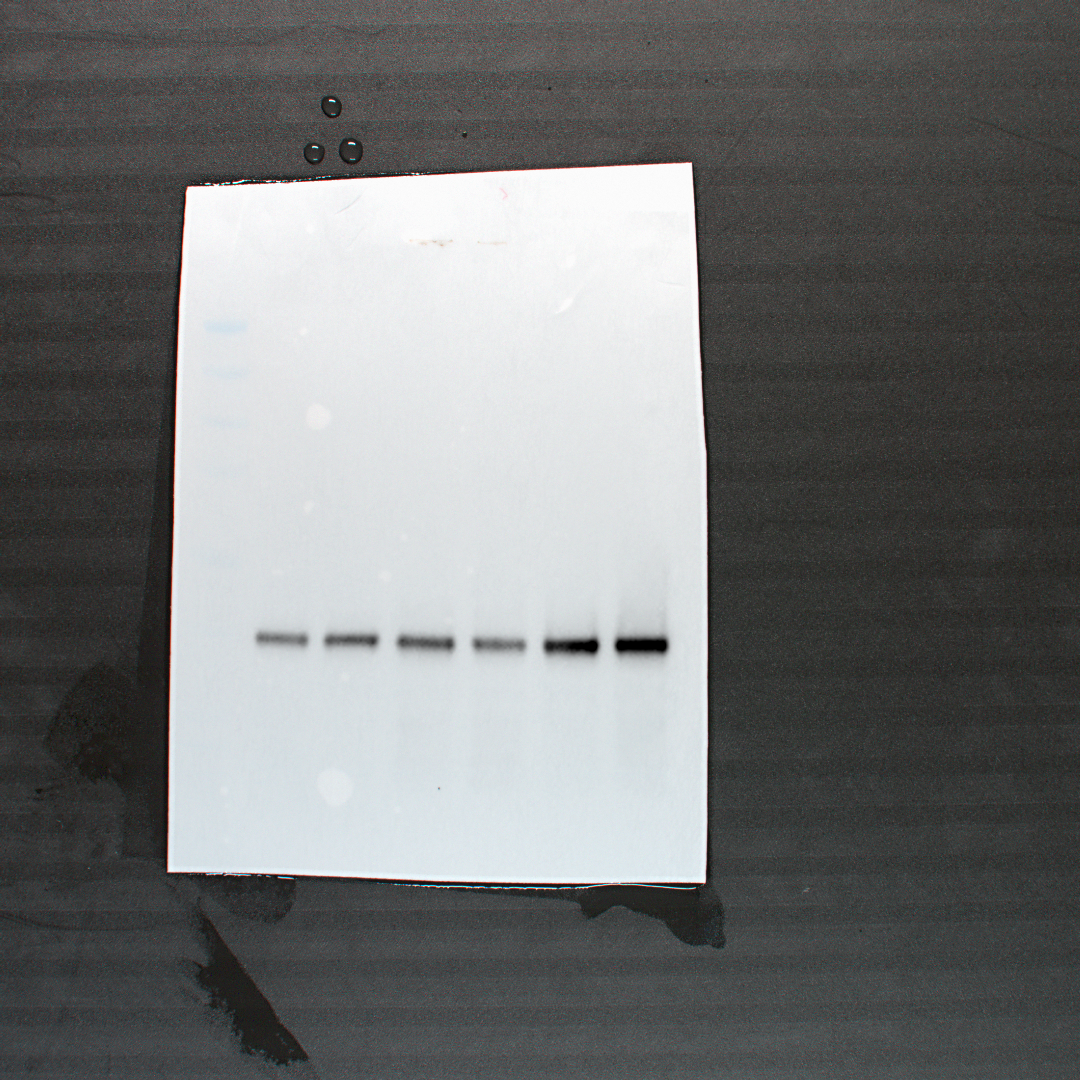

Supplement: Figure 2—figure supplement 1—source data 1. [file elife-86975-fig2-figsupp1-data1.zip › Figure 2-figure supplement1-source data/Figure 2-figure supplement1-source data_a-gapdh_R1.Tif]

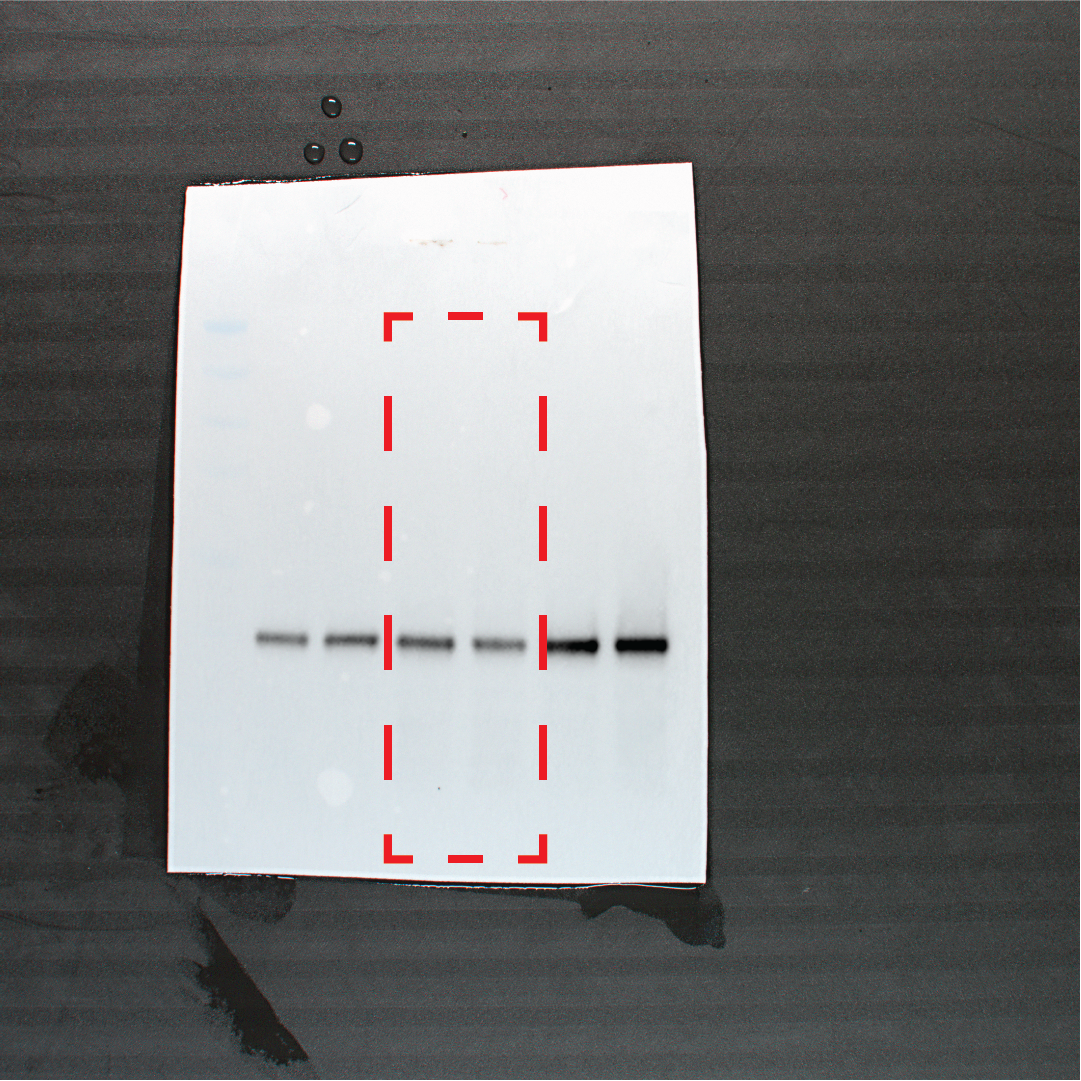

Supplement: Figure 2—figure supplement 1—source data 1. [file elife-86975-fig2-figsupp1-data1.zip › Figure 2-figure supplement1-source data/Figure 2-figure supplement1-source data_a-gapdh_R1_labelled.png]

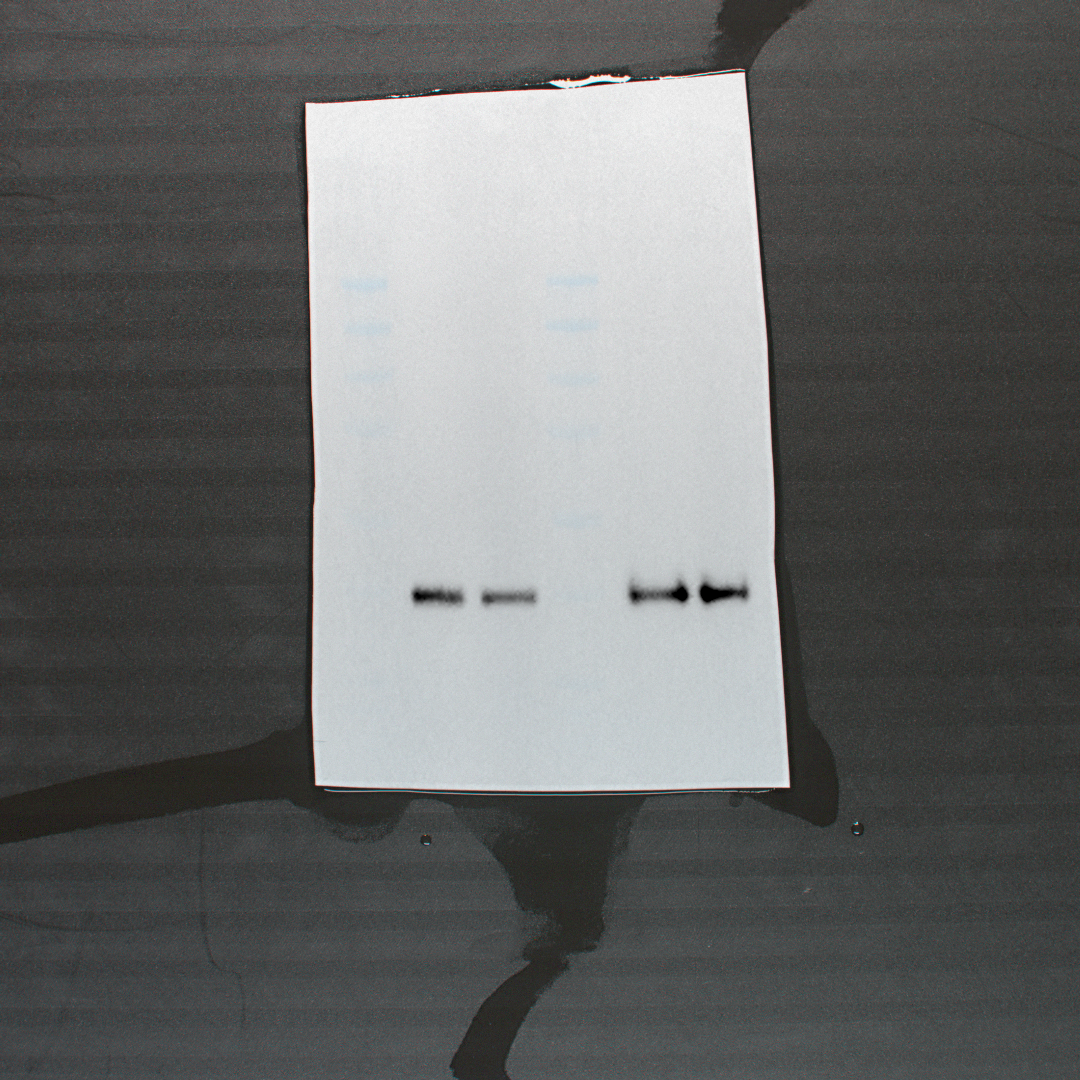

Supplement: Figure 2—figure supplement 1—source data 1. [file elife-86975-fig2-figsupp1-data1.zip › Figure 2-figure supplement1-source data/Figure 2-figure supplement1-source data_a-gapdh_R2.Tif]

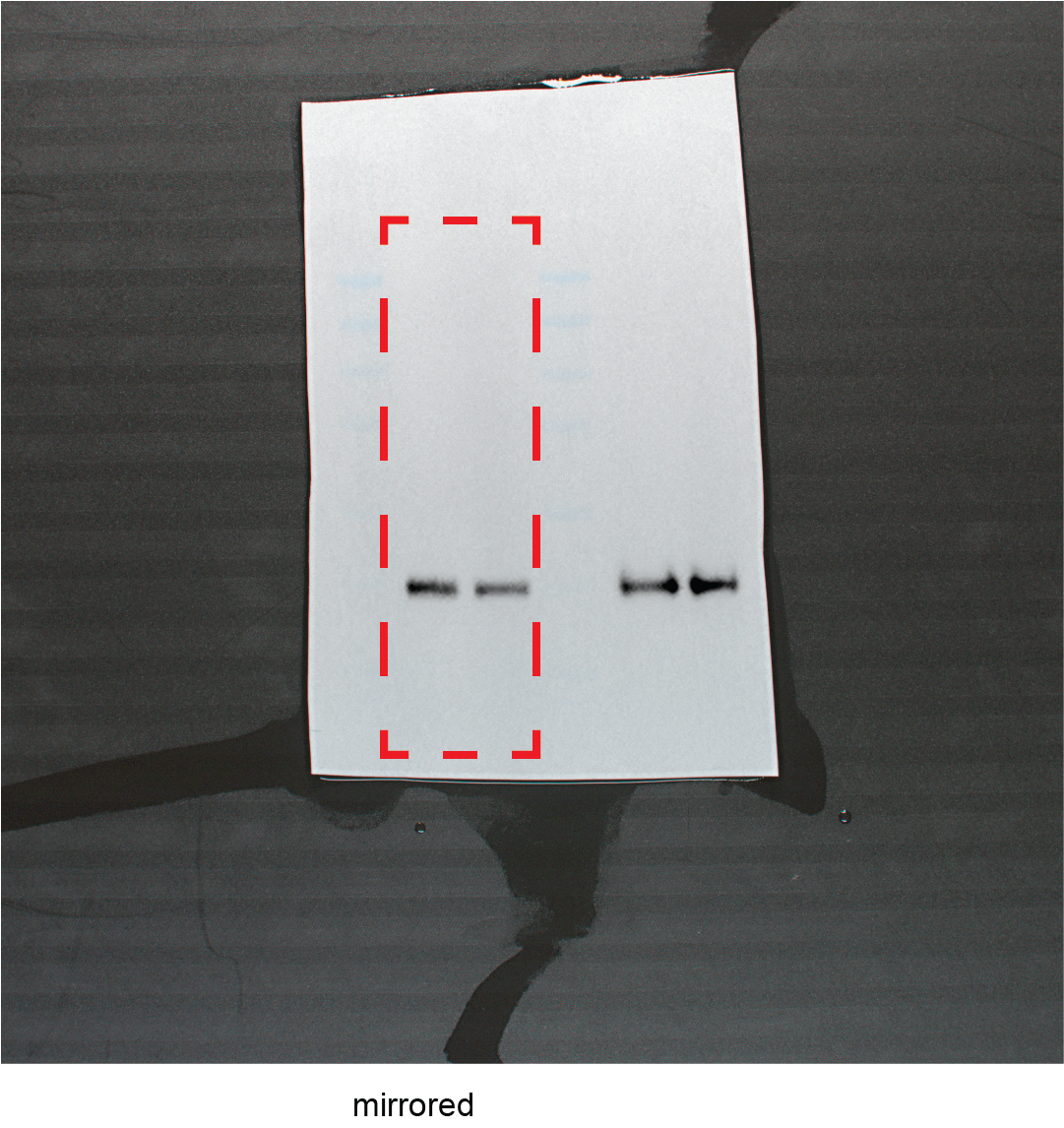

Supplement: Figure 2—figure supplement 1—source data 1. [file elife-86975-fig2-figsupp1-data1.zip › Figure 2-figure supplement1-source data/Figure 2-figure supplement1-source data_a-gapdh_R2_labelled.png]

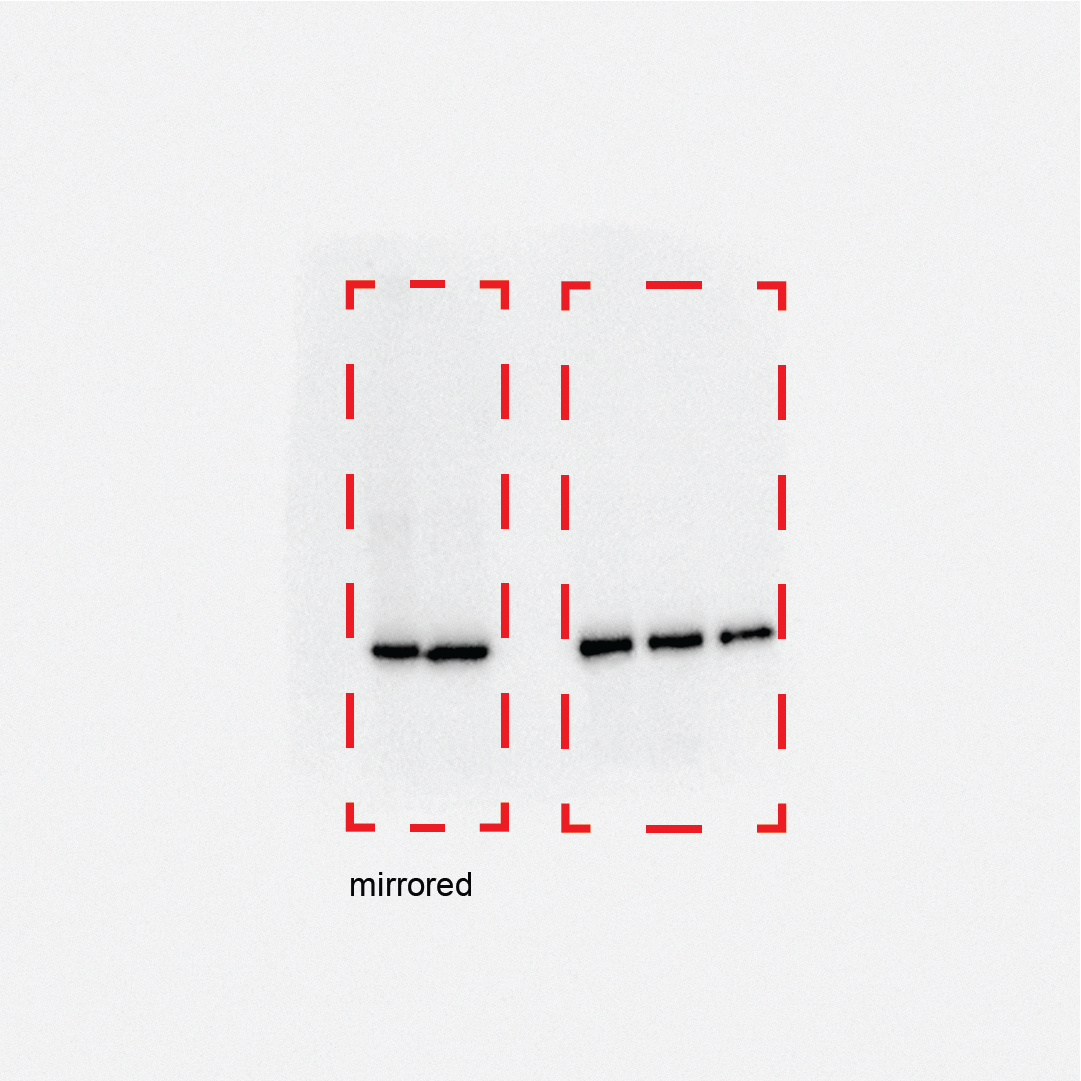

Supplement: Figure 2—figure supplement 1—source data 1. [file elife-86975-fig2-figsupp1-data1.zip › Figure 2-figure supplement1-source data/Figure 2-figure supplement1-source data_a-gapdh_R3+cont_labelled.png]

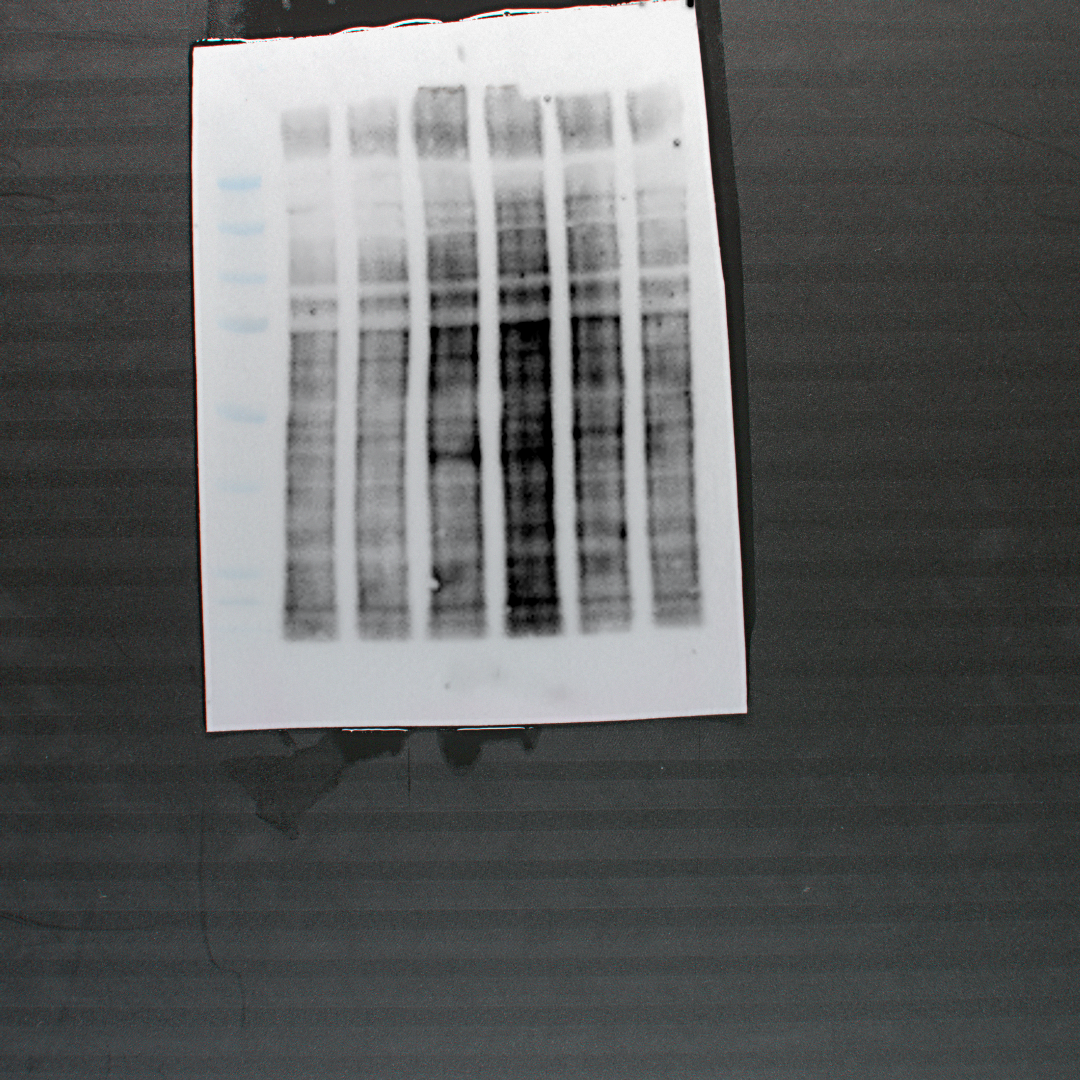

Supplement: Figure 2—figure supplement 1—source data 1. [file elife-86975-fig2-figsupp1-data1.zip › Figure 2-figure supplement1-source data/Figure 2-figure supplement1-source data_a-puro_R1.Tif]

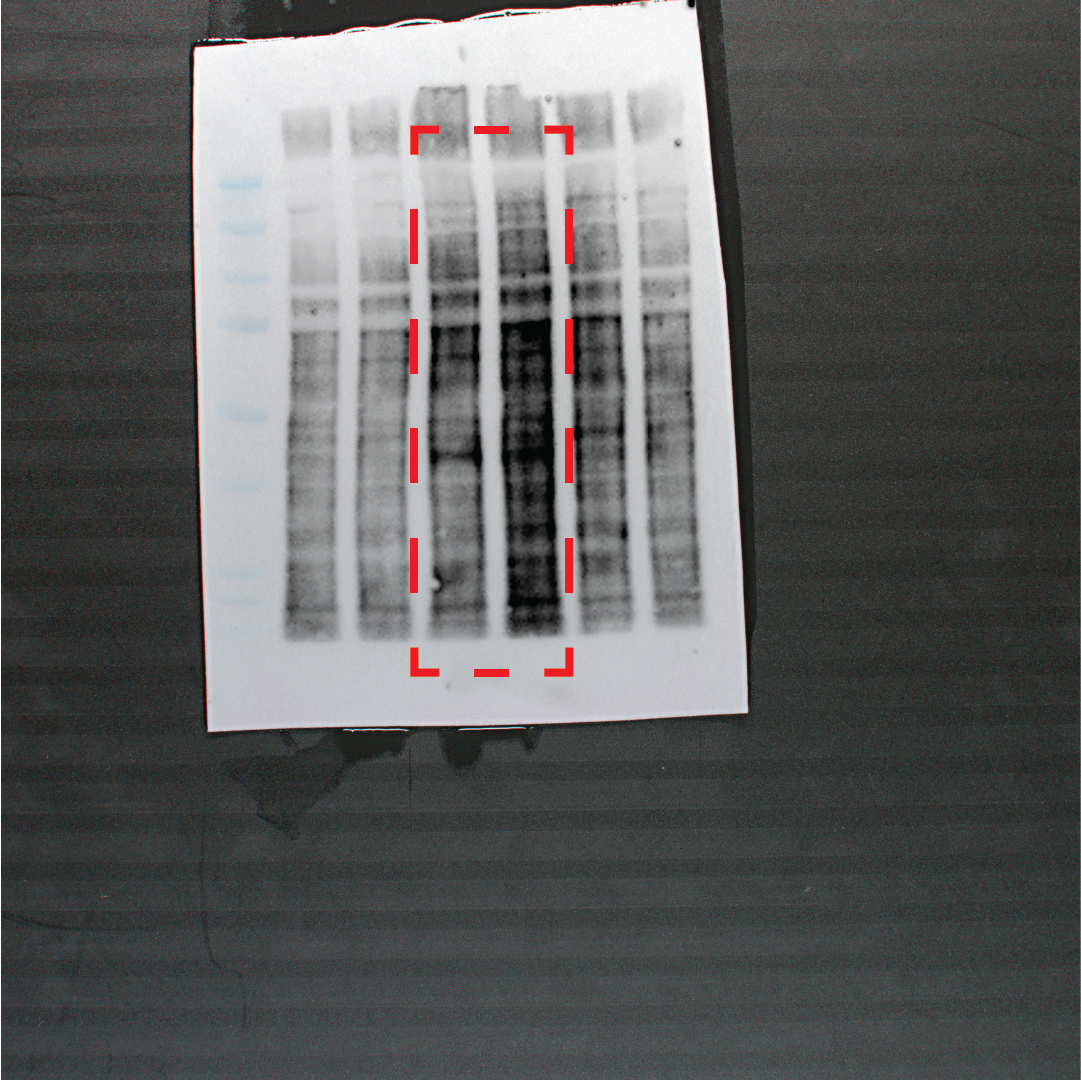

Supplement: Figure 2—figure supplement 1—source data 1. [file elife-86975-fig2-figsupp1-data1.zip › Figure 2-figure supplement1-source data/Figure 2-figure supplement1-source data_a-puro_R1_labelled.png]

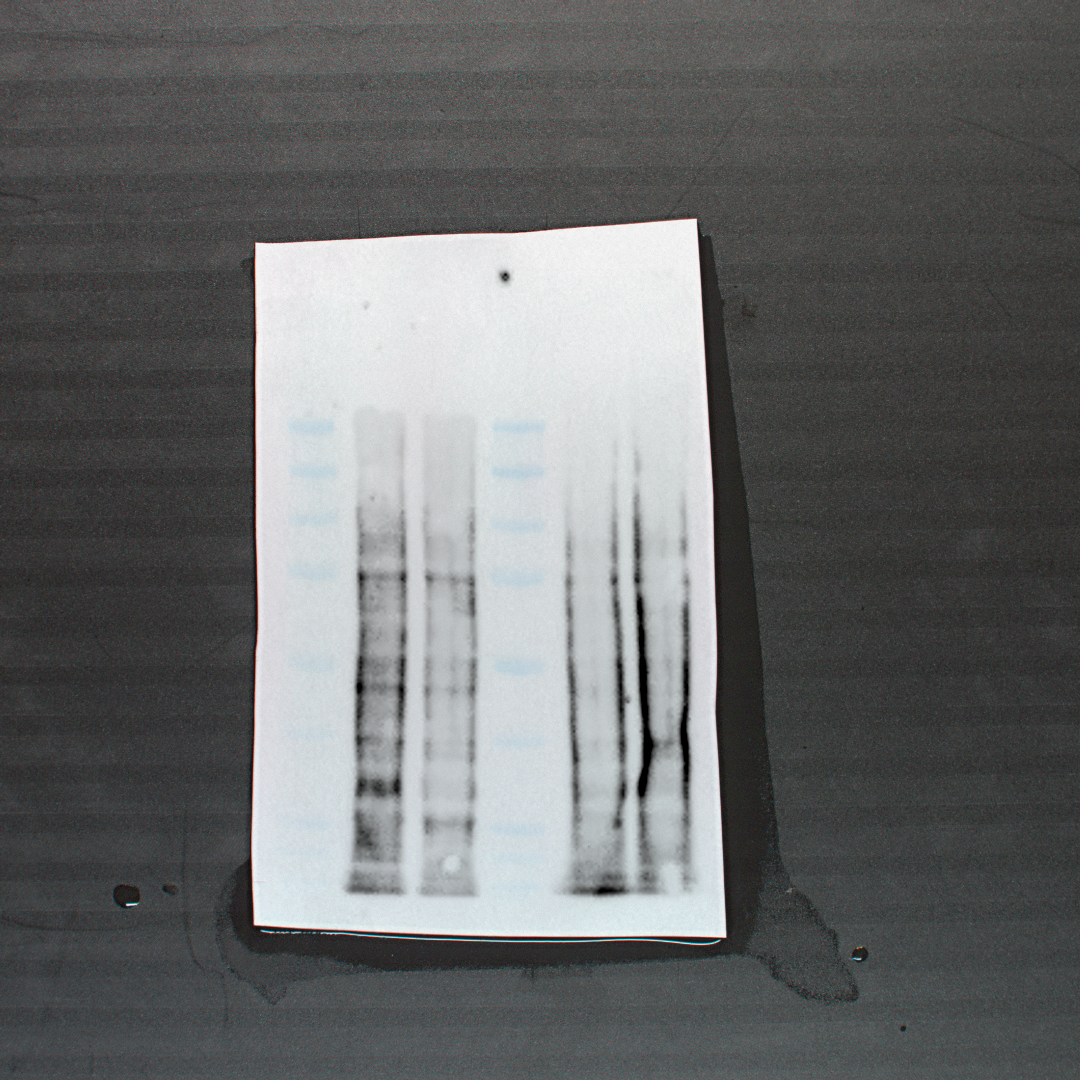

Supplement: Figure 2—figure supplement 1—source data 1. [file elife-86975-fig2-figsupp1-data1.zip › Figure 2-figure supplement1-source data/Figure 2-figure supplement1-source data_a-puro_R2.Tif]

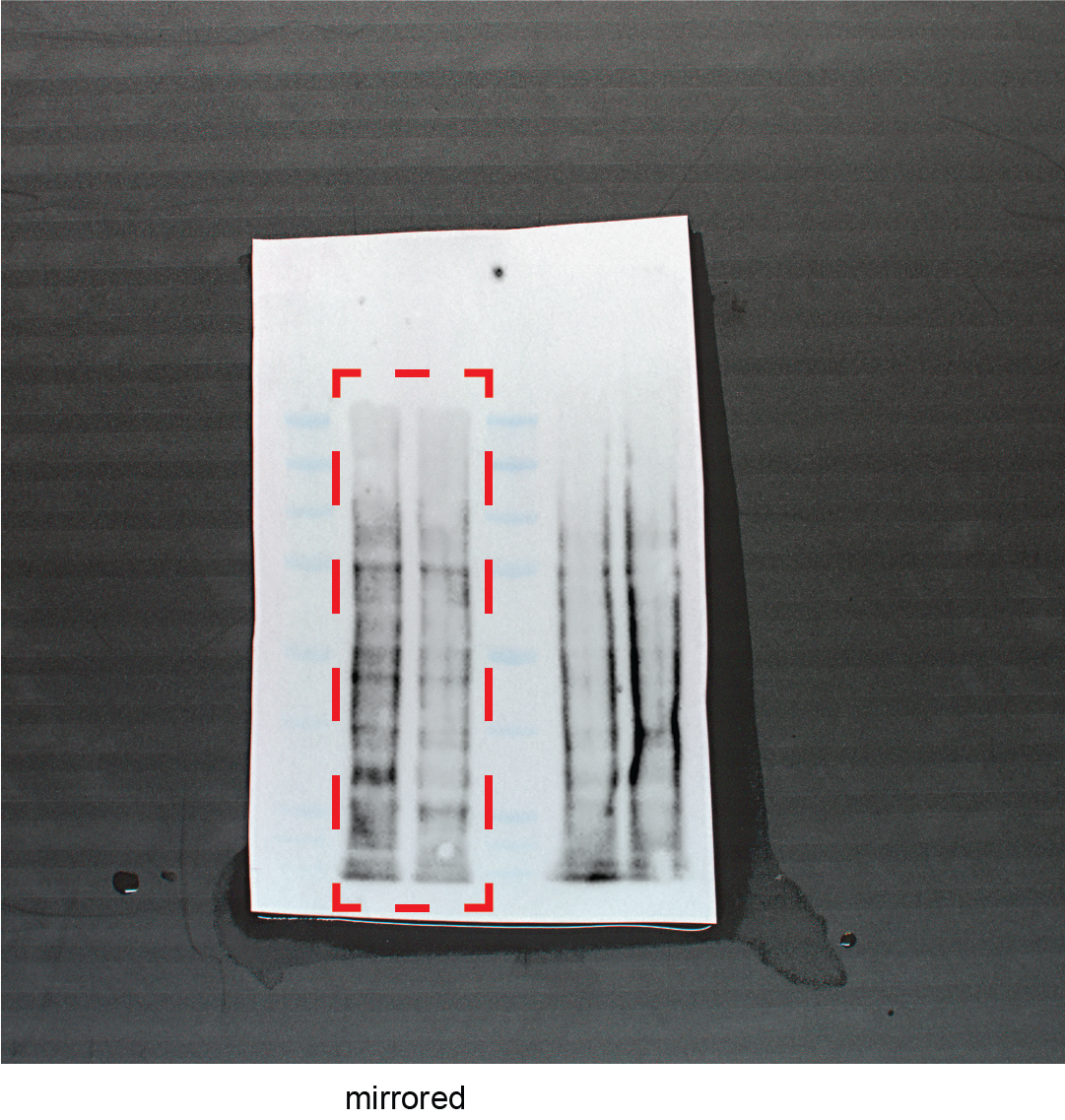

Supplement: Figure 2—figure supplement 1—source data 1. [file elife-86975-fig2-figsupp1-data1.zip › Figure 2-figure supplement1-source data/Figure 2-figure supplement1-source data_a-puro_R2_labelled.png]

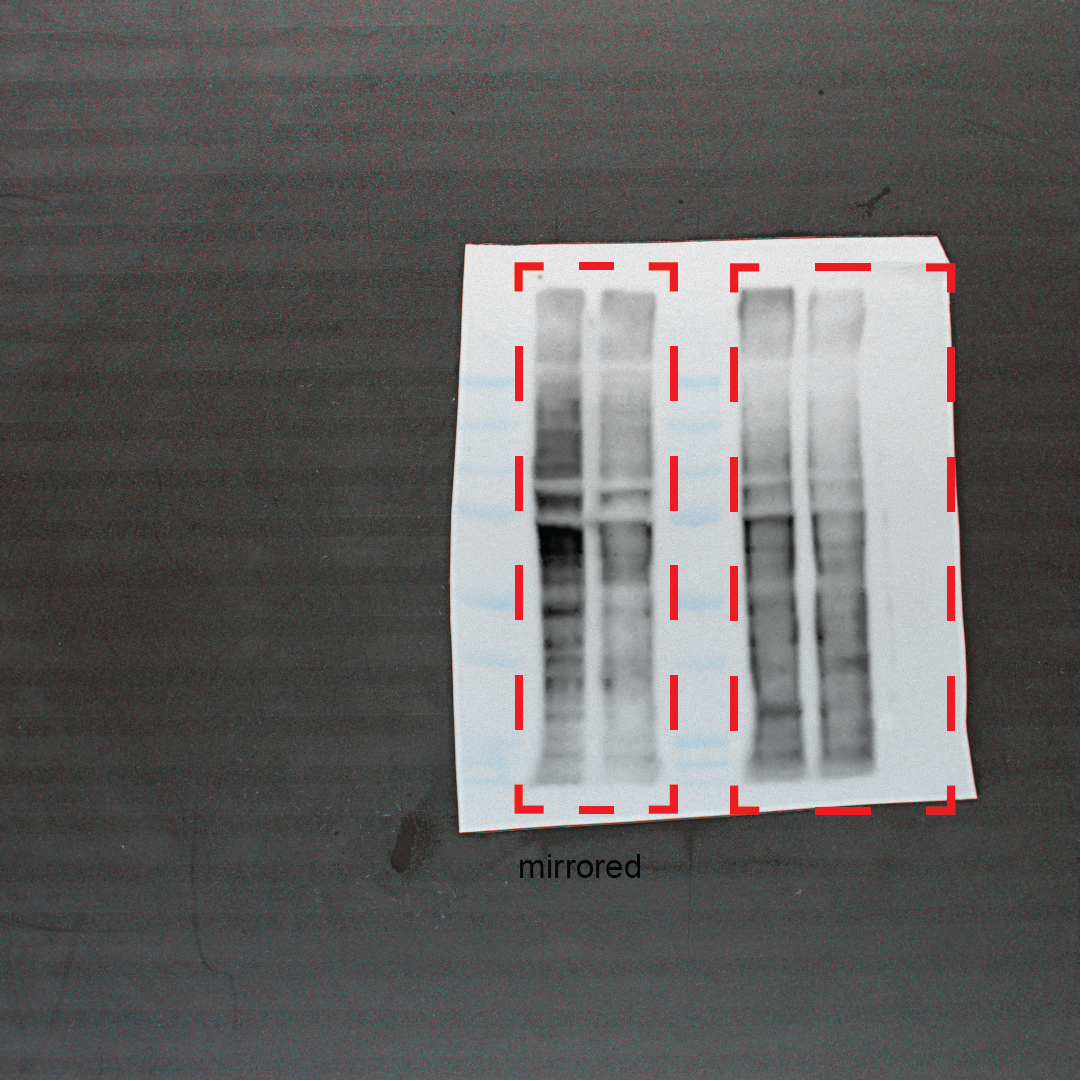

Supplement: Figure 2—figure supplement 1—source data 1. [file elife-86975-fig2-figsupp1-data1.zip › Figure 2-figure supplement1-source data/Figure 2-figure supplement1-source data_a-puro_R3+cont_labelled.png]

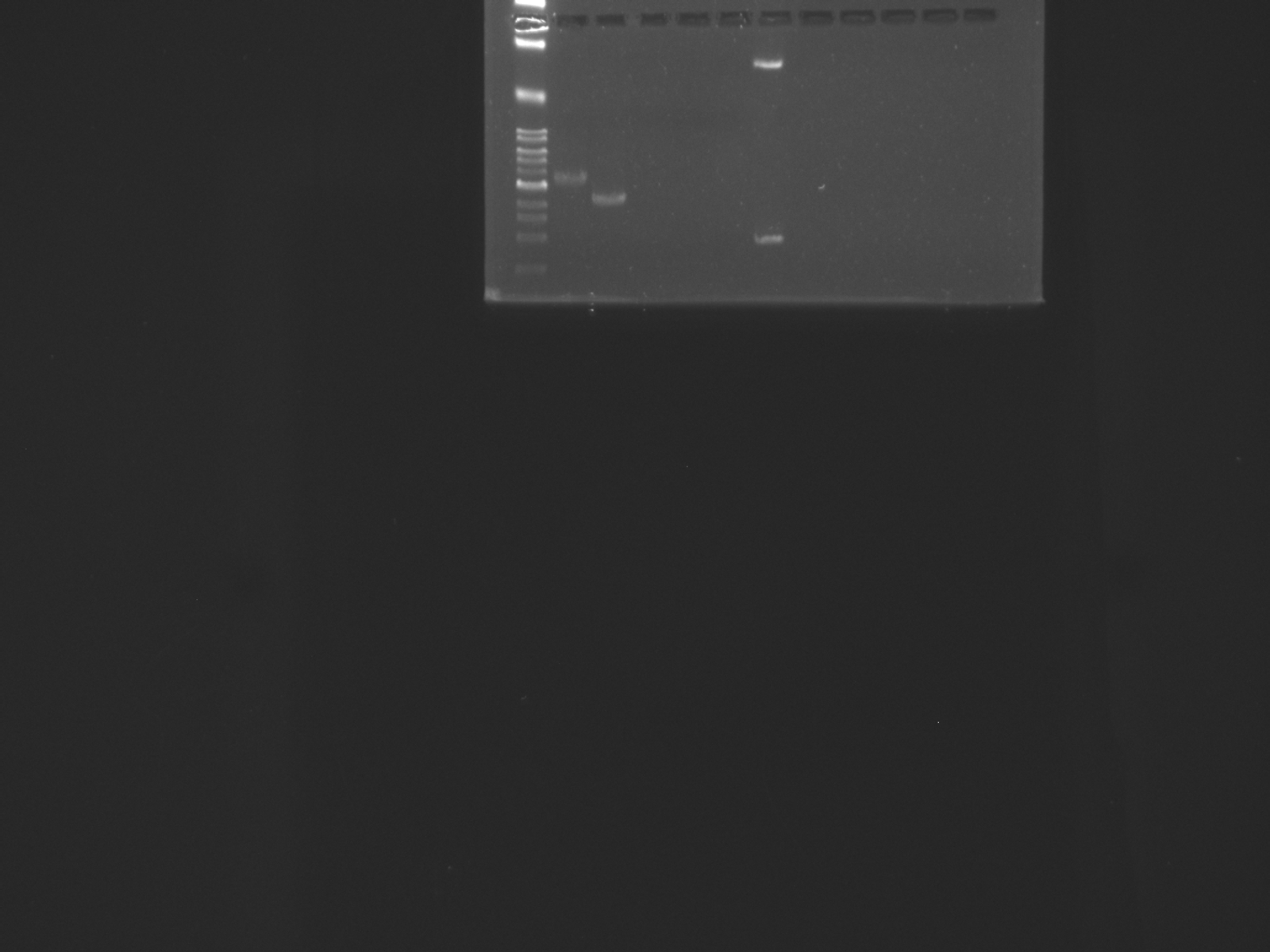

Supplement: Figure 4—figure supplement 3—source data 1. [file elife-86975-fig4-figsupp3-data1.zip › Figure 4-figure supplement 3-source data/Figure 4-figure supplement 3-source data_panel B.Tif]

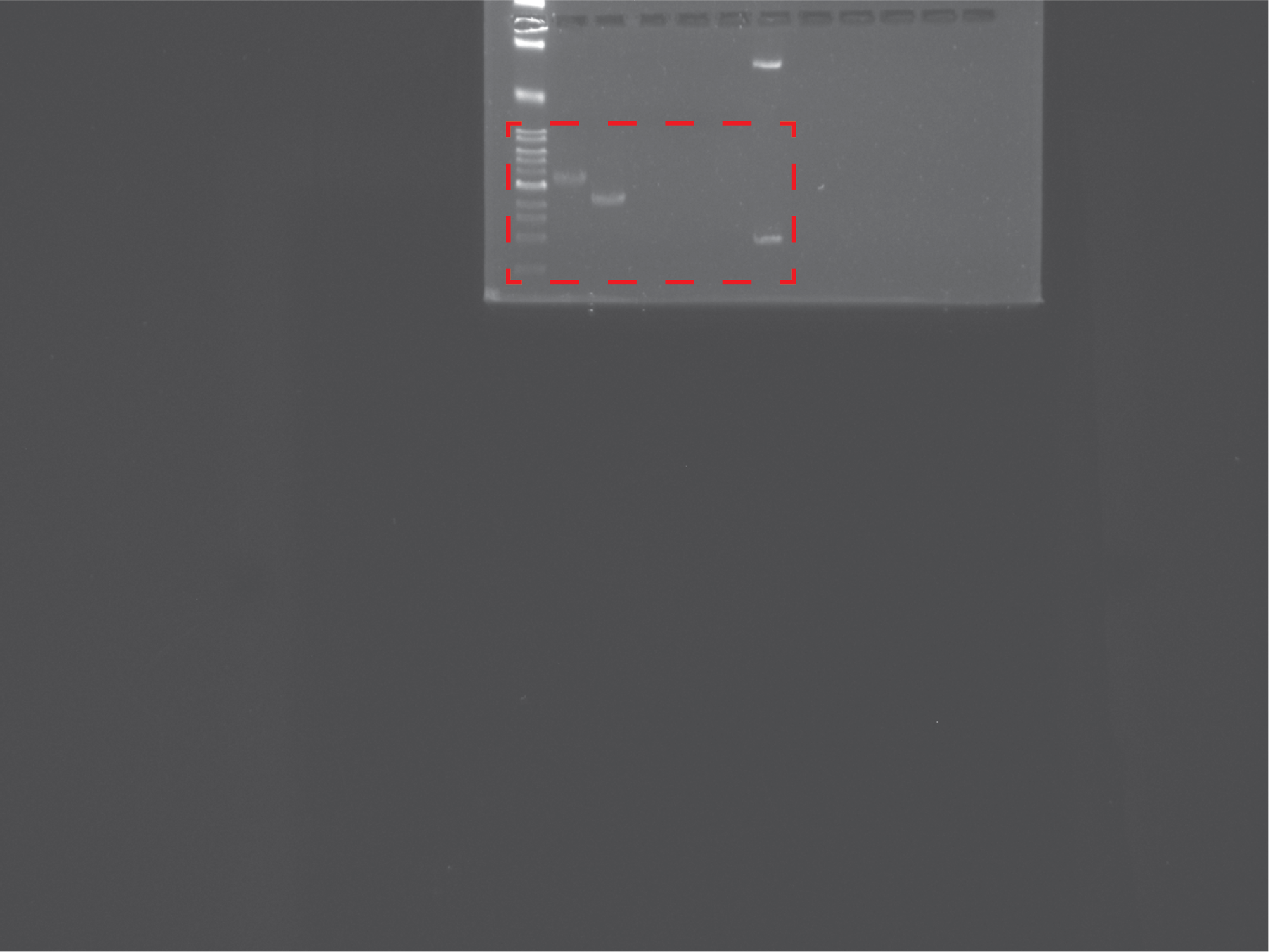

Supplement: Figure 4—figure supplement 3—source data 1. [file elife-86975-fig4-figsupp3-data1.zip › Figure 4-figure supplement 3-source data/Figure 4-figure supplement 3-source data_panel B_labelled.png]

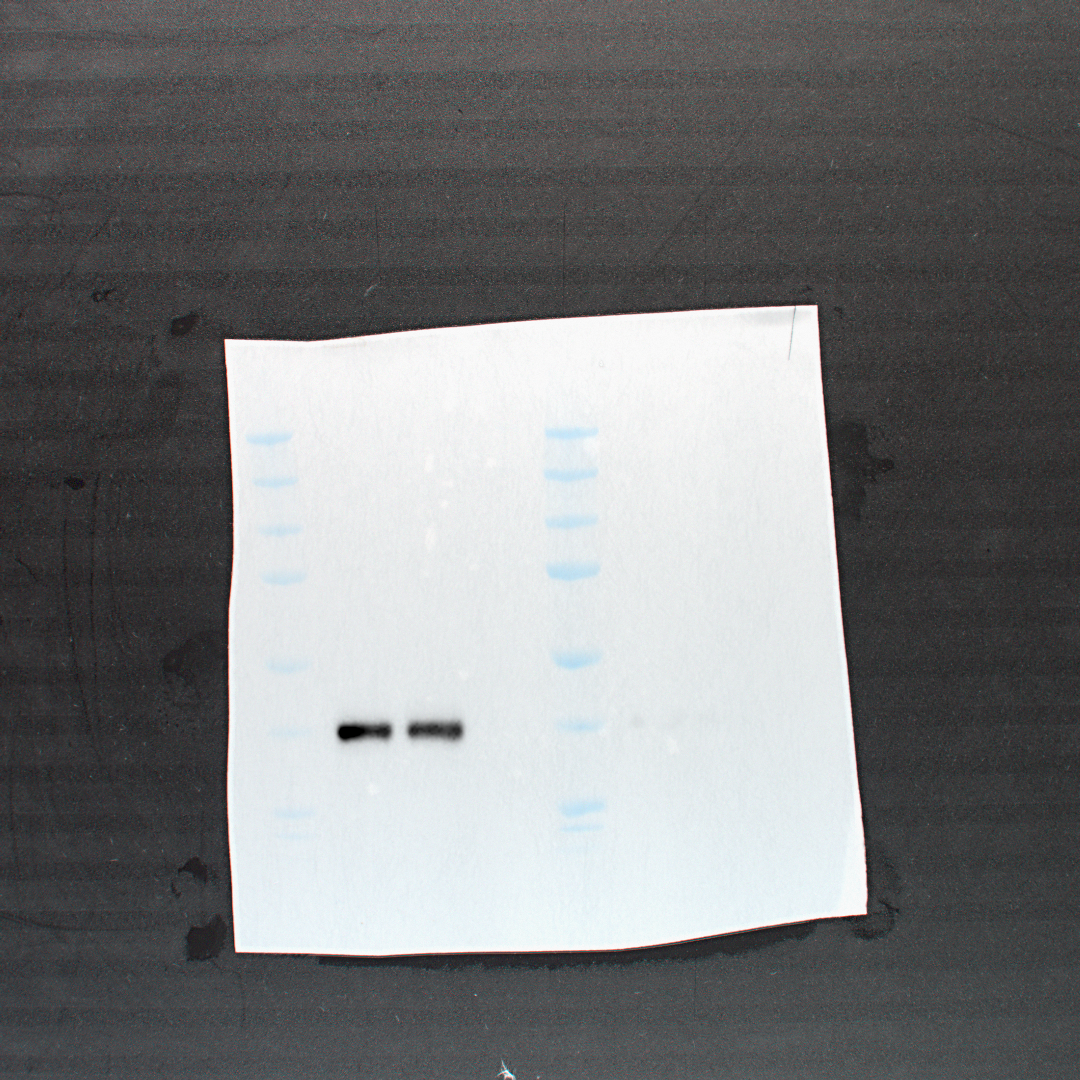

Supplement: Figure 4—figure supplement 3—source data 1. [file elife-86975-fig4-figsupp3-data1.zip › Figure 4-figure supplement 3-source data/Figure 4-figure supplement 3-source data_panel C_a-gapdh_iOE.Tif]

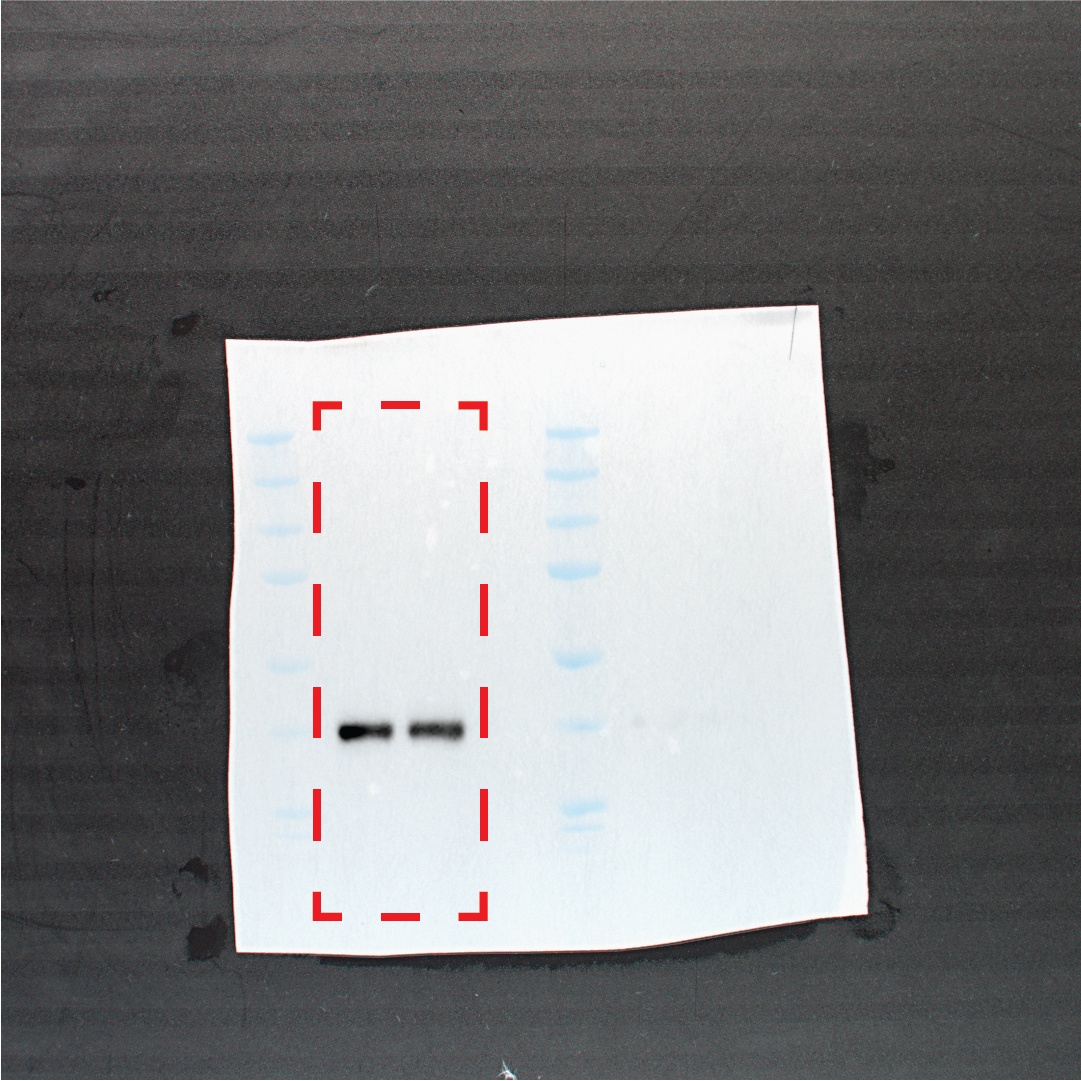

Supplement: Figure 4—figure supplement 3—source data 1. [file elife-86975-fig4-figsupp3-data1.zip › Figure 4-figure supplement 3-source data/Figure 4-figure supplement 3-source data_panel C_a-gapdh_iOE_labelled.png]

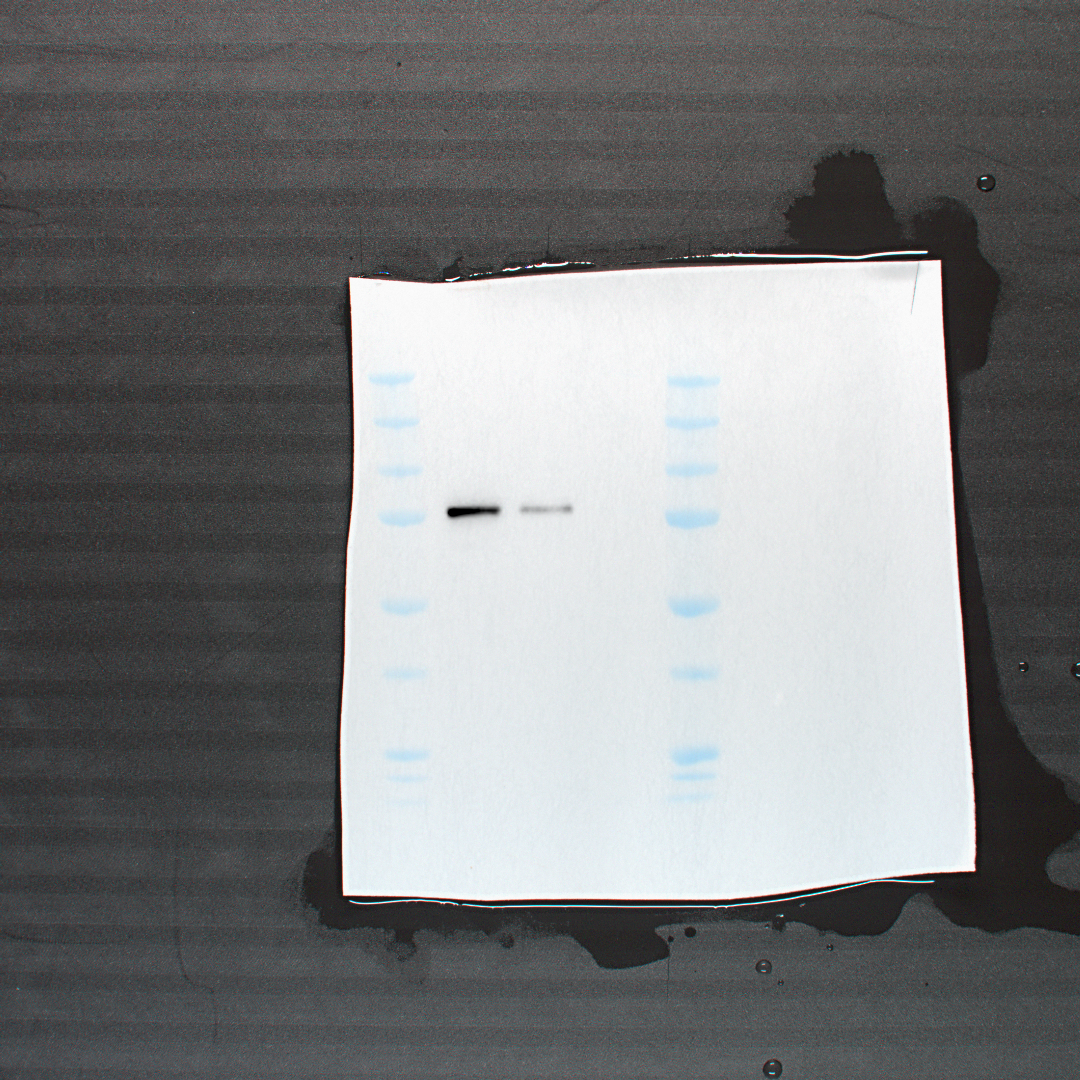

Supplement: Figure 4—figure supplement 3—source data 1. [file elife-86975-fig4-figsupp3-data1.zip › Figure 4-figure supplement 3-source data/Figure 4-figure supplement 3-source data_panel C_a-GFP_iOE.Tif]

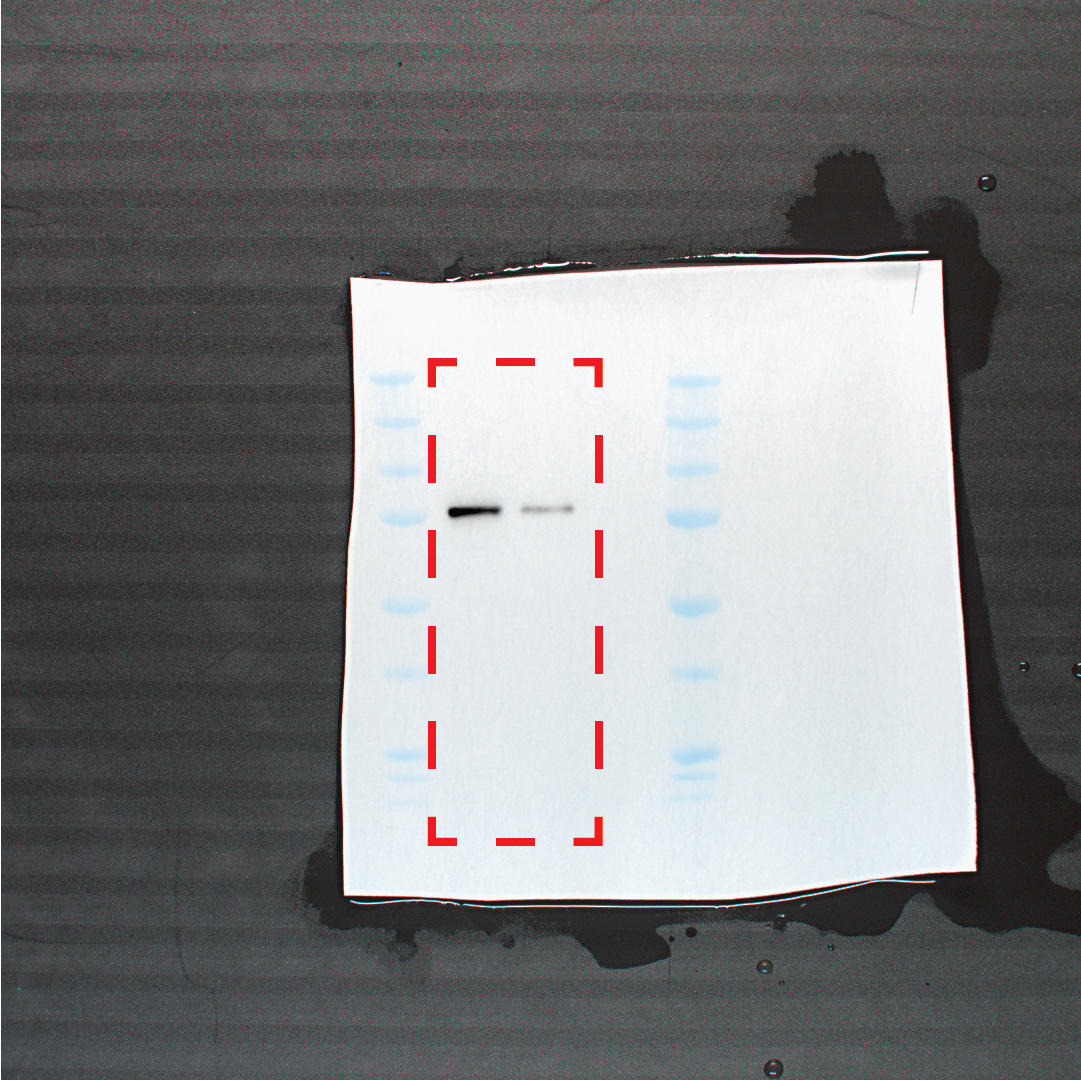

Supplement: Figure 4—figure supplement 3—source data 1. [file elife-86975-fig4-figsupp3-data1.zip › Figure 4-figure supplement 3-source data/Figure 4-figure supplement 3-source data_panel C_a-GFP_iOE_labelled.png]
